# A data and knowledge cross-level fusion-driven learning framework for detecting missing diagnosis

**DOI:** 10.1038/s41746-026-02725-z

**Published:** 2026-05-14

**Authors:** Shaohui Liu, Xien Liu, Xinyue Fang, Chenwei Yan, Kaiyin Zhou, Xinxin You, Meiwei Li, Ji Wu

**Affiliations:** 1 School of Computer Science (National Demonstrative Software School), Beijing University of Posts and Telecommunications, Beijing, China; 2 Key Laboratory of Trustworthy Distributed Computing and Service (BUPT), Ministry of Education, Beijing, China; 3 Department of Electronic Engineering, Tsinghua University, Beijing, China; 4 Tsinghua Shenzhen International Graduate School, Tsinghua University, Guangdong, China; 5 School of Artificial Intelligence and Data Science, University of International Business and Economics, Beijing, China; 6 THiFly Health, Beijing, China; 7 College of AI, Tsinghua University, Beijing, China; 8 Beijing National Research Center for Information Science and Technology, Tsinghua University, Beijing, China

**Keywords:** Computer science, Information technology, Health care economics, Health policy, Health services, Public health

## Abstract

Diagnosis omission in discharge diagnosis lists is common in electronic medical records (EMRs), leading to inaccurate documentation, incorrect Diagnosis Related Group (DRG) assignments, and reduced reimbursements from overlooked Complications and Comorbidities (CC) or Major Complications and Comorbidities (MCC). To address this, we propose a data and knowledge cross-level fusion-driven learning framework for automated identification of missed diagnoses. Evaluated on real-world EMRs from six hospitals across various provinces in China, our model outperforms expert system method, BERT-based method, and multiple LLM-based baseline methods, demonstrating superior F1 scores. Results show 37.8% of EMRs predicted to have missed diagnoses, with 9.0% experiencing altered DRG groupings, subsequently affecting 3.2% of insurance reimbursement. To minimize alert fatigue, we adopted a hybrid approach combining our model with expert system, boosting precision by 6.7–13.4%. We also designed two human-machine coupling modes to demonstrate the utility of our methods in the real world.

## Introduction

Electronic medical records (EMRs) are intended to capture and archive detailed patient treatment information for subsequent medical management and insurance reimbursement^1^. However, the completeness and accuracy of EMRs documentation can be compromised by factors such as physicians’ work-related stress, time constraints, carelessness, and inexperience^2^. As a result, discharge diagnoses recorded in EMRs often face quality issues, including missed diagnoses^3,4^. Studies show missed diagnoses are common, with 27.5% of serious EMR errors across 79 U.S. healthcare institutions were related to missed diagnoses^5^, and 42% of EMRs in 12 Malaysian public healthcare institutions had issues associated with missed diagnoses^3^. Missed diagnoses not only compromise the quality of discharge diagnoses in EMRs but can also impact medical insurance costs due to their effect on Diagnosis Related Group (DRG) payment system. Specifically, the DRG system is a key tool for determining insurance reimbursement by grouping EMRs based on factors such as diagnoses, comorbidities, and complications^6–9^. Therefore, when missed diagnoses lead to missed Complications and Comorbidities (CC) or Major Complications and Comorbidities (MCC), they can directly affect DRG payment amounts. For example, a study in a Melbourne tertiary hospital found that missed additional diagnoses accounted for about 29% of DRG grouping and payment issues^10^. Physicians currently rely on manual detection of missed diagnoses, a time-consuming process due to the complexity and length of EMRs. For example, quickly reviewing a 10-day hospitalization record can take 20–30 minutes, with lengthy documentation increasing the risk of inefficiency and error rates.

Previous research related to EMRs diagnoses has focused on predicting primary diagnoses^11–14^, but rarely takes into account secondary diagnoses and lacks explainability in their results. Although some recent studies have investigated complication prediction or directly predicted DRG based on EMRs to estimate hospital costs using deep learning methods or large language models (LLMs)^15–18^, they often lack the fine-grained ability to identify missed diagnoses. Overall, there is currently a lack of research focused on the automatic detection of missed diagnoses. Therefore, our research aims to identify and rectify missed diagnoses in EMRs, particularly those related to complications or other overlooked conditions, while providing clear evidence for each missed diagnosis.

The task of missed diagnosis detection aims to retrieve confirmed diagnoses present in the hospitalization records that are omitted from the discharge diagnosis list. The input for this task comprises a complete EMR, while the output consists of recommended missed diagnoses. A valid target diagnosis is characterized by two essential criteria: (1) it was deemed clinically confirmed by the attending physician based on available information, and (2) it was absent from the discharge diagnosis list. To meet the requirements of missed diagnosis detection, we propose DKFusion, a data and knowledge fusion learning framework. DKFusion comprises three modules: diagnosis recall, contextual validation, and diagnosis deduplication. The diagnosis recall module rapidly retrieves potential clinical diagnoses from EMRs; the contextual validation module addresses false positives among the retrieved diagnoses; and the diagnosis deduplication module eliminates redundancy between recalled diagnoses and those in the discharge diagnosis list. We enhance the effectiveness of each module by integrating various types of knowledge and data.

**Fig. 1:**
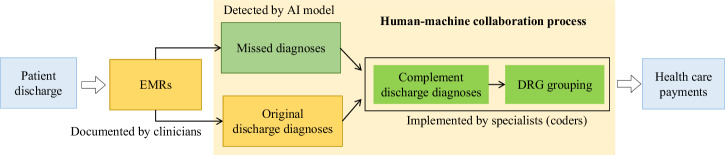
Workflow of the missed diagnosis detection system. The figure illustrates the post-discharge workflow of specialists or coders and shows when the missed-diagnosis detection model enters the human–machine collaboration.

Upon patient discharge, the attending physician finalizes and archives the EMR, generating an initial list of inpatient diagnoses. The EMR is then submitted to the medical affairs department and the medical insurance office, where specialists or coders verify discharge diagnoses and assign corresponding diagnostic codes before the definitive DRG grouping. Our proposed AI-enabled detection model is applied after the patient has been discharged and the physician has completed the full EMR documentation. As illustrated in Fig. 1, the model systematically scans the finalized EMR content to flag potential missed diagnoses. Thereafter, Specialists or coders review both the original discharge diagnoses and the missed diagnoses identified by the AI model. Through this human-AI collaborative workflow, a more complete discharge diagnosis list is established, which subsequently guides accurate diagnosis coding, DRG assignment and insurance reimbursement.

Section “Datasets” provides details of the datasets. In Section “Scenario testing results”, we validated the proposed model’s effectiveness using real-world datasets collected from six hospitals across different provinces and tiers in China. The results demonstrate that DKFusion outperforms traditional baselines and standard LLMs while also achieving comparable F1 performance to task-optimized LLMs with 30B parameters. Section “The impact of missed diagnosis completion on DRG” presents our analysis of missed diagnosis detection’s impact within China’s DRG-based medical insurance payment system using the same datasets. We further conducted comprehensive error analyses in Section “Error analysis” to identify persistent challenges for current diagnostic models. To evaluate clinical applicability, Section “Human–machine collaboration” investigates two distinct human-AI collaboration paradigms adapted to healthcare workflows: the model-driven mode, which prioritizes precision under model leadership with human supervision to minimize prediction errors, and the physician-driven mode, which emphasizes F1, led by specialists with model support to ensure quality control and strike a balance between expert workload and diagnostic integrity.

## Results

### Datasets

We collected 7000 real-world comprehensive EMRs from hospitals to construct the dataset. One EMR represents a single hospitalization admission. It spans six hospitals across five Chinese provinces (Hebei, Anhui, Zhejiang, Jiangsu, and Shanxi), covering northern, central, and southern regions. Specific source information on the data is presented in the Table 1. Of these, the Hebei (HB) dataset comprises 2000 EMRs, while the data from five other hospitals (abbreviated as AH, JS, ZJ, SX-T, and SX-S) each contain 1000 EMRs. This comprehensive dataset serves as the foundation of the study: Firstly, a subset of the data was used to create module-specific training and testing datasets for the individual modules within the DKFusion framework. Secondly, these datasets were independently sampled to construct scenario test sets, covering both in-domain and out-of-domain evaluations, to simulate real-world application scenarios and assess the framework’s overall performance. Finally, the complete dataset was employed for inference and statistical analysis of the missed diagnosis rate in real-world scenarios. The following paragraphs detail the training and testing sets for the modules, as well as the scenario testing datasets used in this study.

**Table 1:** Source and specifics of the data

Data	Province	Region	Hospital level	City development status	Medical insurance payment	Year of collection
HB	Hebei	Northern China	Tertiary care hospital	Municipal city	Fee-for-service	2020
AH	Anhui	Central China	Tertiary care hospital	Provincial city	DRG payment	2023
ZJ	Zhejiang	Southern China	Tertiary care hospital	Municipal city	DRG payment	2022
JS	Jiangsu	Central China	Tertiary care hospital	Municipal city	Fee for service	2022
SX-T	Shanxi	Northern China	Tertiary care hospital	Municipal city	Fee for service, DRG payment	2023
SX-S	Shanxi	Northern China	Secondary care hospital	County city	Fee for service,DRG payment	2023

**Fig. 2:**
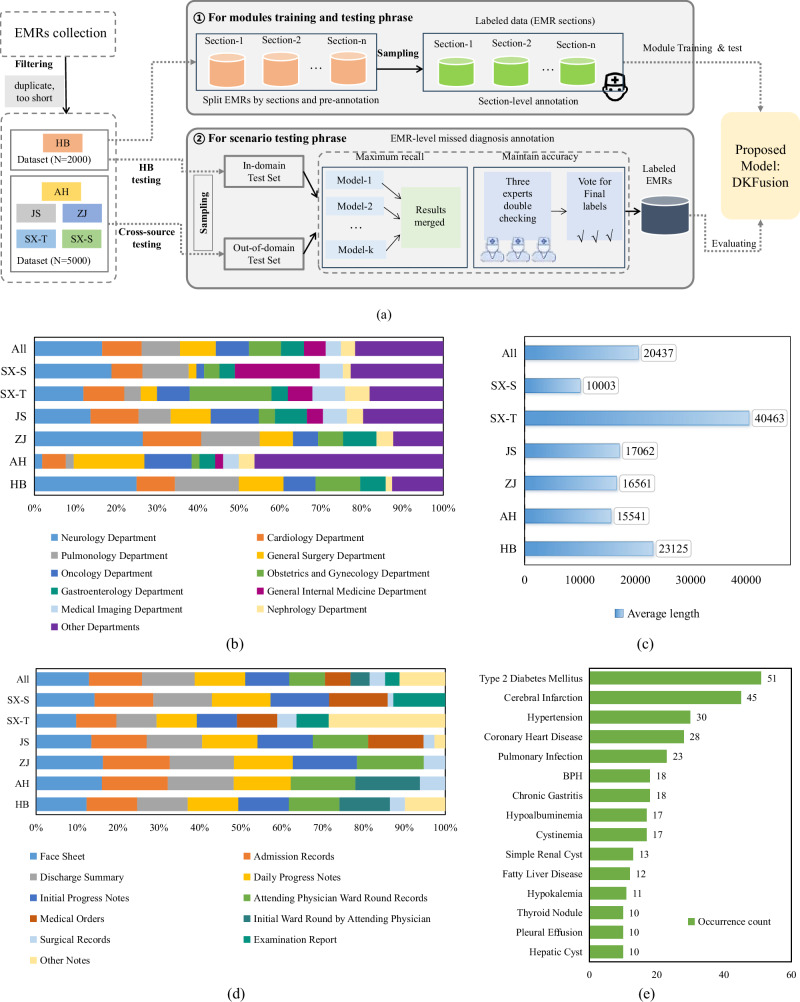
Data annotation and statistical analysis of the annotated test set. **a** The datasets annotation and construction workflow for the modules training and testing phase and scenario testing phase. **b** The distribution of departments across different hospitals. **c** The average token length of EMRs at various hospitals. **d** The distribution of clinical notes counts among the hospitals. **e** The top 15 most frequently occurring diagnoses across the entire test set, along with their respective frequencies.

To streamline the training and testing of individual modules, we constructed customized datasets tailored to the section-level functional requirements of each DKFusion submodule, rather than processing entire EMRs. We implemented the pipeline illustrated in Fig. 2a to ensure efficient dataset construction while mitigating the label imbalance often found in random sampling approaches. First, EMRs were segmented to align with their semi-structured format and specific module objectives: the diagnosis recall module targeted specific clinical sections (e.g., “present illness”); the contextual diagnosis validation module extracted the target diagnosis alongside its preceding and succeeding sentences; and the diagnosis deduplication module paired candidate diagnoses with discharge diagnoses entries. Next, we employed heuristic rule-based pre-annotation tools and label-distribution sampling to assist physicians in systematically selecting and annotating samples. Finally, the validation and deduplication submodules utilized expert-annotated datasets from the HB repository (comprising 9925 and 4999 labeled instances, respectively, split for training/testing), while the diagnosis recall module employed a dictionary-based approach evaluated exclusively on test data (yielding 1813 diagnosis mentions from 482 instances). Complete details regarding dataset composition, label distribution, and annotation methodologies for each module are provided in Table 2.

**Table 2:** Overview of annotation dataset composition

Type	Number	Annotation strategy	Annotator	Label distribution
Modules training and testing phase				
Diagnosis recall	Clinical sections: 482	Direct Annotation	Mid level doctor	diagnosis mentions (1813)
Contextual validation	Diagnosis-contexts: 9925	Direct Annotation	Mid level doctor	confirmed (5662), unconfirmed (3604), uncertain (659)
Diagnosis deduplication	Diagnosis pairs: 4999	Direct Annotation	Mid level doctor	similar (1031), included (1710), secondary (274), irrelevant (1984)
Scenario testing phase				
In-domain Test set	EMRs: HB(62)	Annotation and voting	3 mid level doctors	Number of miss diagnosis: HB (129)
Out-of-domain test set	EMRs: AH(51), ZJ(49), JS(51), SX-T(50),SX-S(53)	Annotation and voting	3 mid level doctors	Number of miss diagnosis: AH (199), ZJ (255), JS (191), SX-T (122), SX-S (145)

During the scenario testing phase, we sought to construct a test set by annotating missed diagnoses across entire EMRs. However, manual annotation of lengthy EMRs is both labor-intensive and error-prone, with manual efforts often failing to capture all missed conditions. To mitigate this, we adopted a “human-machine complementary” strategy to establish a more reliable gold standard with reduced manual effort, as illustrated in Fig. 2a. The methodology follows four key phases: (1) Multi-method prediction, where baseline models and the DKFusion method generate a comprehensive list of potential missed diagnoses; (2) Result aggregation, which consolidates these predictions to ensure maximum coverage; (3) Expert annotation, involving three experts (blinded to the prediction source) who independently validated each candidate against clinical documentation; and (4) Integration, where final labels were determined via majority voting. This process yielded an approximated gold standard for comprehensive missed diagnoses per EMR. In total, 1812 candidate missed diagnoses were annotated and evaluated. The three physicians demonstrated substantial inter-rater agreement (Fleiss’ Kappa *K* = 0.751, 95% CI: 0.720-0.782), with limited inconsistencies attributed primarily to variations in clinical experience and subjective interpretation of the labeling criteria.

Finally, for the in-domain test set, 62 EMRs were sampled from the complete HB dataset, with the physician annotations used to evaluate general physician performance. Selection prioritized on maximizing EMR completeness and ensuring realistic, broad departmental coverage. The out-of-domain test set was used to assess model generalization. Due to resource constraints, physicians were not involved in the multiple-method prediction step. We retained EMRs where at least one method predicted missed diagnoses, while improving department coverage and EMR completeness. In the final out-of-domain test set, the sample sizes sampled from AH, ZJ, JS, SX-T, and SX-S are 51, 49, 51, 50, and 53. Some statistical results related to the scenario test set are depicted in Fig. 2. Regarding department distribution (Fig. 2b), the dataset spans various specialties, prioritizing common departments while retaining diversity. The Neurology Department (16.6%), Cardiology Department (9.7%), and Pulmonology Department (9.4%) are the most represented, while departments outside the top 10 collectively account for 21.6% of the sample. Regarding text volume (Fig. 2c), the EMRs exhibit significant variation in length, consistent with real-world clinical data. The dataset shows a mean length of 20,437 tokens and a median of 16,543 tokens, with a substantial standard deviation of 16,473 (Range: 3940–141,371). Regarding the composition of clinical notes (Fig. 2d), Admission Records, Face Sheets, and Discharge Summaries emerge as the three most predominant types, accounting for 13.0%, 13.0%, and 12.9% of the total note volume, respectively. Crucially, these core documents are present in nearly all EMRs, reflecting the completeness of the notes in the test set. Meanwhile, beyond the 10 most common types, various other note types collectively account for the remaining 11.2%. Figure 2(e) presents the 15 most common diagnoses in the test set. Table 2 presents the annotated number of missed diagnoses in the in-domain and out-of-domain test sets, which varies among hospitals. Notably, the out-of-domain test set uses EMRs with missed diagnoses predicted by at least one method to enable comprehensive missed diagnosis analysis, but artificially inflates the rate, which does not reflect real-world prevalence.

### Scenario testing results

**Fig. 3:**
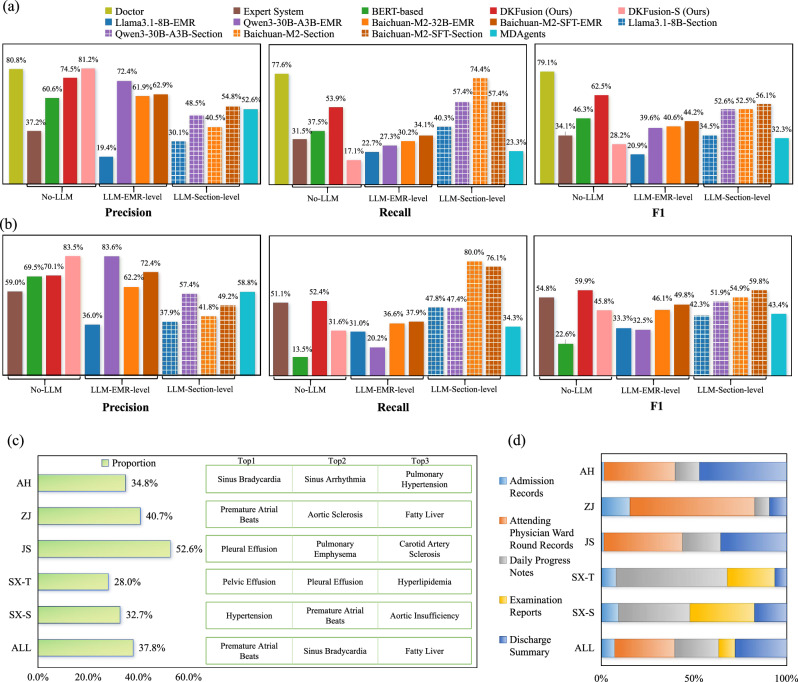
Performance of different models on the scenario testing, with the proportion and source distribution of missed diagnoses within the EMRs. **a** The experimental results from in-domain test (HB=62). **b** The average experimental results of different models across various out-of-domain datasets (AH=51, ZJ=49, JS=51, SX-T=50, SX-S=53). **c** The proportion of EMRs with missed diagnoses predicted by the DKFusion model across the different dataset (AH=1000, ZJ=1000, JS=1000, SX-T=1000, SX-S=1000), along with the most frequently missed diagnoses in each dataset. **d** The distribution of missed diagnoses predicted by the model across different clinical notes. The tested LLMs (Llama3.1-8B, Qwen3-30B-A3B, Baichuan-M2-32B) underwent EMR-level evaluation by processing entire EMR for direct end-to-end missed diagnosis prediction. Alternatively, we evaluated a section-level approach, where clinical sections were processed individually before merging and deduplicating partial predictions to generate the final EMR-level results.

To evaluate the framework’s performance in detecting missed diagnoses in real-world EMR scenarios, we used in-domain (same source as module training) and out-of-domain (five other hospitals) test sets. A mid-level physician manually annotated the in-domain data for comparison, while we also tested recall-verify frameworks like Expert Systems, BERT-based methods, and various LLM configurations. Specifically, to comprehensively evaluate LLMs capabilities, we tested three different types of advanced models (Llama3.1-8B^19^, Qwen3-30B-A3B^20^, and Baichuan-M2-32B^21^) across different granularities (section-level and end-to-end) and optimization strategies (e.g., supervised fine-tuning (SFT) and multi-agent setups). Performance was measured using precision, recall, and F1 score, with F1 as the primary metric for balanced assessment. Results are shown in Fig. 3.

The performance of the physicians in Fig. 3 suggests that, when reviewing the entire EMR, mid-level physicians may face challenges in comprehensively and accurately detecting missed diagnoses, achieving a precision of 80.8% and a recall of 77.6%. This underscores the inherent complexity of the task: physicians must carefully examine the EMR from start to finish, cross-referencing documented diagnoses with the discharge diagnosis list to detect omissions. The process requires frequent backtracking through records, which not only increases the likelihood of errors but also proves highly time-consuming. DKFusion demonstrates superior performance across both in-domain and out-of-domain settings, substantial surpassing traditional baselines (including BERT-based and expert-system approaches), as well as LLMs utilizing standard instruction prompting or general medical multi-agent frameworks (such as MDAgents^22^). Specifically, DKFusion achieved F1 scores of 62.5% on the in-domain set and 59.9% on the out-of-domain set. Even when compared to LLMs utilizing targeted supervised SFT, DKFusion remains highly competitive; it outperforms the second-best model, Baichuan-M2-SFT-Section, by 6.4% on in-domain tasks while maintaining comparable out-of-domain generalization (+0.1%). Overall, while LLMs show immense potential in this task when subjected to targeted optimization, our model achieves greater efficiency and robustness through a fusion-driven strategy of knowledge and data. It accomplishes this despite having less than 1% of the parameter count of Baichuan-M2-32B and lacking the extensive world knowledge derived from massive pre-training. Furthermore, given the strict computational and security constraints that prevent most hospitals from locally deploying 30B-parameter models or accessing external APIs, DKFusion offers a highly practical and accessible solution for clinical deployment.

Through experiments with different LLM configurations on the missed-diagnosis detection task, We observed several important findings: (1) Section-level vs. EMR-level strategy: Section-level models consistently outperformed their EMR-level counterparts (e.g., Baichuan-M2-SFT-Section achieved 56.1% (in-domain) and 59.8% (out-of-domain) vs. 44.2% and 49.8% for EMR-level). When using the EMR-level strategy, LLM processing complex and lengthy EMRs often exhibit information loss (e.g., “lost-in-the-middle^23^"), making the model more conservative by retaining only highly reliable answers. This results in higher precision but lower recall rates. In contrast, the section-level strategy processes EMRs in chunks, reducing information omission and boosting recall. However, lacking global context and insufficient task-specific knowledge, LLMs often fail to distinguish between incidental clinical mentions and valid discharge diagnoses required for DRG coding. This deficiency leads to frequent false positives, include disease risks (e.g., arrhythmia susceptibility), normal physiological findings (e.g., sinus rhythm), procedures (e.g., tracheal intubation), and frequent diagnosis deduplication failures (e.g., pulmonary infection vs. pneumonia). (2) Importance of domain adaptation: The medical-adaptation LLM Baichuan-M2 outperformed general LLMs in most of experiments, with task-specific SFT further boosting F1 scores. Specifically, the section-level Baichuan-M2-SFT achieved F1-score improvements of 3.6% (from 52.5% to 56.1%) on in-domain data and 4.9% (from 54.9% to 59.8%) on out-of-domain data compared to the base Baichuan-M2, while End-to-end approaches exhibited similar gains. (3) Performance of general medical agents: General LLM-based multi-agent frameworks, such as MDAgents, demonstrated poor performance (F1 scores of 32.3% in-domain and 43.4% out-of-domain). We observed that while MDAgents autonomously recruits multiple expert agents with different backgrounds, each individual agent remains constrained by a lack of specific understanding regarding this task and an inability to fully comprehend the complete EMR. Furthermore, complex agent interactions can introduce additional errors during information transmission^24^, rendering these frameworks ineffective at addressing the missed diagnosis detection task.

To estimate the prevalence of missed diagnoses in EMRs, we used the DKFusion model to predict across datasets from different hospitals (AH=1000, ZJ=1000, JS=1000, SX-T=1000, SX-S=1000). Figure 3c displays the predicted missed diagnosis rates across datasets, with JS having the highest rate (52.6%) and SX-T the lowest (28.0%), averaging 37.8%. Through discussions with physicians and an analysis of the correlation between DRG implementation duration and missed diagnosis rates across hospitals, we observed a notable inverse trend that longer adoption periods correlate with lower missed diagnosis rates (Pearson r = −0.92, as shown in Supplementary Fig. 9). Despite the limited sample size, the contrast between the extremes is striking. For instance, SX-T, which adopted DRG in 2021(2.0 years), exhibited the lowest missed diagnosis rate (28.0%). In contrast, JS, which still operates under a fee-for-service model(0 years), showed the highest rate (52.6%). These findings suggest that DRG payment adoption may be a pivotal factor–when hospitals are reimbursed via DRG, they develop a clear financial incentive to minimize diagnostic omissions. This mechanism likely encourages hospitals to recognize the importance of diagnostic accuracy and enforce stricter management protocols.

To better understand the causes of missed diagnoses, we analyzed their sources within clinical notes. As illustrated in Fig. 3d, most missed diagnoses originate from superior physician rounds, daily progress notes, and discharge summaries. From a documentation-timing perspective, records from superior physician rounds are typically completed post-rounds, during which superior physicians often clarify and confirm multiple diagnoses. Similarly, daily progress notes document abnormalities and diseases identified during rounds. Through discussions with physicians, we identified that the continuous documentation demands in these two note types–coupled with heavy clinical workloads–may cause physicians to inadvertently overlook non-primary diagnoses. Such omissions often result in these diagnoses being excluded from the final discharge diagnoses, representing a key contributor to missed diagnoses.

We conducted a comprehensive analysis of the per-institution out-of-domain test sets (detailed in Table 3). DKFusion consistently outperformed the expert system and the BERT-based method, achieving higher precision, recall, and F1 scores across most institutions. Compared to Baichuan-M2-32B-SFT, which represents the top LLM-based methods we tested for this task, DKFusion generally exhibits superior precision but lower recall. Consequently, F1 scores varied by institution: hospitals AH and SX-T favored DKFusion, while JS and SX-S favored Baichuan-M2-32B-SFT, resulting in comparable average performance between the two approaches. From the results, we found that the same model exhibited variations in performance across different datasets, and most models performed relatively poorly on the SX-T dataset. To analyze this interesting cross-hospital variability, we examined five out-of-domain test sets using four key documentation indicators: (1) Field Completeness (coverage ratio of 14 core EMR sections), (2) Repetition Rate (copy-paste frequency measured by edit distance), (3) Negative Symptom Ratio (a proxy for linguistic complexity), and (4) Discharge Diagnostic Completeness. By normalizing and combining these metrics into a composite complexity score, we identified a negative correlation with DKFusion’s performance (*r* = −0.842; Supplementary Fig. 8). This association suggests that performance degradation is not random but systematically linked to specific EMR attributes–namely, high text repetition, linguistic complexity, noise-laden clinical descriptions, and stringent institutional protocols regarding diagnostic completeness. These factors explain why the SX-T dataset yielded the lowest performance. Two characteristics were particularly influential: First, SX-T had the highest discharge diagnostic completeness (73.7%), leaving only subtle, hard-to-identify missed diagnoses. Second, additional sections such as “Differential Diagnosis" in SX-T introduced substantial noise, challenging models’ ability to extract key signals from complex EMR text. Details of the experiments are provided in Supplementary Section 3.7.

**Table 3:** Comparison of model performance (%) across different out-of-domain test sets

Model	AH			ZJ			JS			SX-T			SX-S		
	P	R	F	P	R	F	P	R	F	P	R	F	P	R	F
Expert System	71.2	51.8	60.0	61.9	50.0	55.3	55.5	49.3	52.2	39.0	50.6	44.0	72.0	54.5	62.0
BERT-based	75.0	15.0	25.0	74.1	15.3	25.4	66.6	10.6	18.2	56.4	14.2	22.7	71.8	11.6	19.9
Llama3.1-8B-EMR	34.4	36.2	35.3	38.2	25.9	30.9	37.6	32.5	34.8	22.4	21.3	21.8	46.0	39.3	42.4
Q3-30B-A3B-EMR	78.0	16.1	26.7	81.3	20.4	32.6	85.7	15.7	26.5	80.6	23.8	36.7	93.2	28.3	43.4
BM2-EMR	60.1	41.7	49.2	65.5	37.3	47.5	68.1	31.4	43.0	44.0	27.0	33.5	69.2	43.4	53.4
BM2-SFT-EMR	72.3	36.7	48.7	66.9	31.0	42.3	88.2	47.1	61.4	56.1	37.7	45.1	77.3	40.0	52.7
Llama3.1-8B-Sec	37.8	45.2	41.2	45.2	41.2	43.1	46.0	53.9	49.6	21.5	49.2	29.9	44.1	53.8	48.5
Q3-30B-A3B-Sec	67.5	38.7	49.2	61.2	47.1	53.2	60.7	38.7	47.3	40.0	57.4	47.1	62.8	62.8	62.8
BM2-Sec	32.9	61.8	42.9	52.3	81.2	**63.6**	53.7	80.6	64.5	27.5	90.2	42.1	46.9	93.8	62.5
BM2-SFT-Sec	46.2	63.3	53.4	58.3	67.5	62.5	60.8	85.3	**71.0**	31.8	86.9	46.6	52.7	87.6	**65.8**
MDAgents	55.5	33.2	41.5	63.4	40.0	49.1	66.3	30.9	42.1	46.7	35.2	40.2	60.6	29.7	39.8
DKFusion	74.0	58.5	**65.4**	68.1	50.6	58.1	67.5	52.6	59.1	56.4	45.4	**50.3**	85.2	52.5	65.0
DKFusion-S	88.2	31.3	46.2	75.6	28.0	40.9	90.4	34.6	50.1	73.3	28.5	41.1	88.7	35.8	51.0

The indicators P, R, and F represent precision, recall, and F1. Among these, BM2 is the abbreviation for Baichuan-M2-32B, Q3 stands for Qwen3, and EMR and Sec respectively indicate EMR-level and section-level testing. The values in bold represent the highest F-score within the same dataset.

In Hospital Information System (HIS) integrations, where the model autonomously alerts physicians and coders to potential missed diagnoses, high precision is critical to mitigating alarm fatigue–a well-documented phenomenon where excessive false alerts lead to desensitization and increased oversight of critical notifications^25–27^. To address this, we developed DKFusion-S, a precision-optimized DKFusion variant for scenarios demanding higher accuracy in real-world applications. The precision performance of the DKFusion-S model is illustrated in Figs. 3(a) and (b). According to the results, the DKFusion-S model improves precision by 6.7—13.4% compared to DKFusion. The performance across different datasets, as shown in Table 3, further supports the consistent precision advantage of DKFusion-S. As demonstrated in Fig. 3(a), DKFusion-S achieves comparable precision to physicians (81.2% vs. 80.8%) on the in-domain test set (HB). A comparison of the model’s precision across different clinical departments is provided in Supplementary Fig. 7, which reveals that DKFusion-S exhibits certain advantages in Neurology, Oncology, and Surgery departments. Notably, under the default EMR-level configuration, Qwen3-30B-A3B achieved comparable average precision to DKFusion-S on the out-of-domain test set (83.6% vs. 83.5%). However, its F1-score was significantly lower (32.5%) compare to DKFusion-S (45.8%), indicating that to attain similar precision, it sacrificed substantially more recall compared to DKFusion-S.

### The impact of missed diagnosis completion on DRG

DRG is currently the popular healthcare payment system worldwide, and China is actively promoting a payment system based on DRG^28^. In the six different datasets described earlier, the hospitals involved have either adopted or are in the process of adopting DRG grouping as their reimbursement method. To assess the impact of missed diagnoses on DRG grouping-especially with respect to our model’s predictions-we performed this analysis across all in-domain and out-of-domain test sets from six regions (total n=316). We used our study in version 1.1 of CHS-DRG (China Healthcare Security Diagnosis Related Groups), which defines 628 groups using China’s medical insurance ICD (the International Classification of Diseases)-10 (v2.0) diagnosis codes and ICD-9-CM-3 surgical codes, integrating international DRG principles with adaptations to Chinese clinical and insurance data. A detailed description of DRG version can be found in Supplementary Section 3.1.

The specific experimental procedure is as follows: (1) Input of these data into our DKFusion-S yielded results identifying missed diagnoses, which were then coded using ICD. The encoding is performed using a predefined diagnosis-to-ICD code mapping table, as referenced in Section “Dictionary-based diagnosis recall.” (2) These diagnoses were evaluated for their status of CC/MCC and compared against the original DRG groups. Discrepancies in the levels of CC / MCC prompted revisions to the DRG groups. This procedure generated DRG groupings adjusted for missed diagnoses, as illustrated in the Supplementary Fig. 5. It is important to note that missed diagnosis detection will only result in either no change or an elevation of the original grouping (for example, adjusting a case from “without complications" to “with general complications") and will not lead to a reduction in the grouping.

**Fig. 4:**
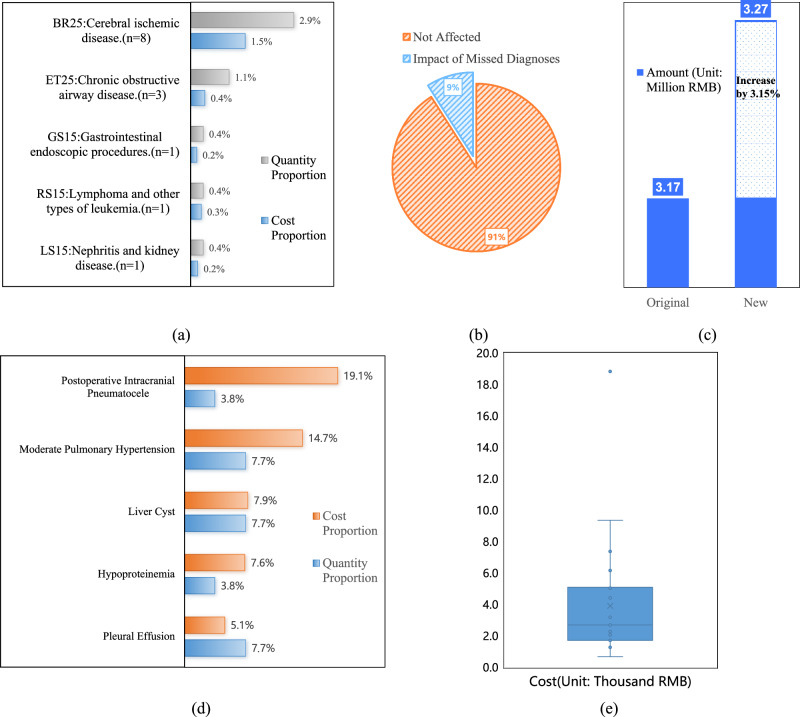
An analysis of the impact of missed diagnosis costs within the CHS-DRG grouping system. Experiments conducted across all in-domain and out-of-domain test sets from six regions (total n=316). Missed diagnoses were predicted by the DKFusion-S model, and the grouping costs were sourced from official documents published by the Anhui (AH) Medical Insurance Bureau. We calculated the number of affected groupings and the proportion of impacted costs among all the EMRs predicted to contain missed diagnoses, along with various statistical metrics. To mitigate the influence of outlier cases, we excluded the two EMRs that had the highest and lowest impact of missed diagnoses on costs. **a** The top five DRG groups encountered in the test set, along with their associated costs. **b** Among the EMRs with missed diagnoses, the proportion that impacted DRG grouping. **c** The percentage of cases that affect DRG grouping, leading to increased costs. **d** The top five missed diagnoses that have the greatest impact on costs. **e** Summarizes the statistical metrics related to these cost influences.

Figure 4 presents the detailed experimental results. The findings indicate that in the DRG payment system, 9.03% of the cases with missed diagnoses found by our model will lead to DRG grouping changes, resulting in an increase of 3.15% in medical insurance payments. This emphasize the significant impact of missed discharge diagnoses within the DRG payment system. We also found that the impact of missed diagnoses on costs varies significantly, related to CHS-DRG settings and the original DRG grouping of the EMRs. For medical groups with high original costs, missed diagnoses may have a more significant impact on the care process, leading to a greater impact on costs when CC/MCC is added. For example, the outlier presented in Fig. 4e illustrates a case where a missed diagnosis resulted in a financial loss of 18,849 RMB. The case involved a patient with malignant intracranial tumors who developed postoperative intracranial pneumatocoele following tumor resection. In this EMR, the intracranial pneumatocoele affected postoperative recovery, complicating the recovery process and prolonging hospitalization. Its omission as a CC diagnosis from the discharge diagnosis list resulted in incorrect grouping and financial loss.

### Error analysis

We conducted a detailed error analysis of the results, as shown in Table 4, identifying two main categories of errors: false positives and false negatives. The EMR texts in the table were translated from the original Chinese data.

**Table 4:** Case of error analysis

Case	Error type	EMRs / Discharge diagnosis	Recalled diagnose
False positives (29.9%)			
Case 1	False positives in context	Invited department: Ophthalmology ...Consultation purpose: The patient has a newly diagnosed case of diabetes. Please consult to examine the fundus to clarify the condition of **retinal disease**.	**Retinal disease**
Case 2	Diagnosis already recorded	Pregnancy complicated by frequent premature ventricular contractions (PVCs)	**Arrhythmia**
False negativates (47.6%)			
Case 1	Recall failures	Following admission, the patient underwent comprehensive diagnostic workup, with brain MRI revealing a sellar space-occupying lesion, accompanied by secondary **suprasellar cistern herniation** and mild midbrain compression, though without evidence of acute hydrocephalus. Neurosurgical consultation concluded ...	**Suprasellar cistern herniation**
Case 2	Erroneous clinical associations	Old cerebral infarction	**Multiple cerebral infarction**

Content translated from chinese EMRs.

False positives refer to incorrectly identified missed diagnoses, primarily occurring in two scenarios: (1) False positives in context: The patient does not actually have the predicted missed diagnosis during current admission. (2) Diagnoses already recorded in discharge diagnoses: Predicted missed diagnoses are duplicated with discharge diagnoses. Typical error cases are illustrated in Table 4. While designing the framework and annotating data, we focused heavily on addressing these two issues. However, the diversity of the EMRs means that the model cannot learn all instances of false positive diagnoses. Upon investigation, we discovered that these errors arise from specific instances within the test data. For instance, in Case 1, a retinal disease is potentially confirmed by the relevant department but still requires validation from a consultative department, making it a subtle situation that is challenging for the model to grasp. In addition, when deduplicating candidate diagnoses against recorded discharge diagnoses, although we optimized our approach by infusing extensive related knowledge, fully understanding all diagnostic interactions remains an significant challenging task.

False negatives in our system primarily manifest through two distinct pathways: (1) Recall failures, where the diagnosis recall module misses some diagnoses due to limitations in the supported diagnosis dictionary (e.g., suprasellar cistern herniation in Case 1 was undetected as it wasn’t included in the predefined list); and (2) Erroneous clinical associations, where the model incorrectly links diagnoses that are similar in wording but distinct in clinical meaning (e.g., in Case 2, newly developed multiple cerebral infarctions were incorrectly associated with a pre-existing old cerebral infarction, despite these being medically independent diagnoses that should both be recorded separately).

While the DKFusion model incorporates specialized design and demonstrates superior performance compared to baseline approaches, several limitations remain noteworthy. The observed false positive and false negative rates of 29.9% and 47.6%, respectively, indicate significant room for improvement in the model’s capabilities.

### Human–machine collaboration

**Fig. 5:**
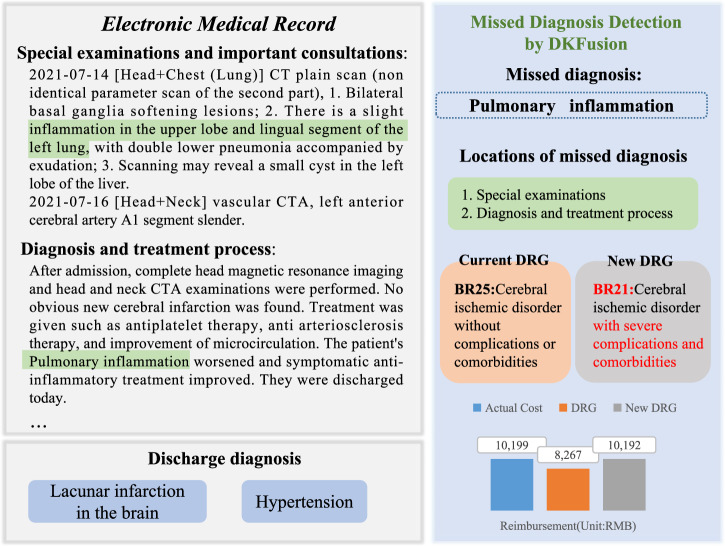
Schematic diagram of human–machine interaction with the missed diagnosis detection system. Physicians can quickly locate multiple instances of missed diagnoses within the EMRs simultaneously upon receiving the results of the missed diagnosis detection, facilitating rapid identification and decision-making. This interactive diagram also demonstrates the interpretability of our proposed DKFusion framework.

Compared to typical end-to-end deep learning models, such as those used for diagnostic prediction and ICD coding, which often lack interpretability beyond the results, physicians using these tools are required to review the entire EMR to validate the accuracy of the predictions. This verification process demands considerable time and effort, making such systems less user-friendly for doctors. In contrast, our designed missed diagnosis detection framework provides task-specific interpretability. Not only does it predict missed diagnoses, but it also highlights multiple locations of the missed diagnoses within the EMR as evidence, facilitating quick confirmation by the physician. An example of interaction with the missed diagnosis detection system is illustrated in Fig. 5.

When considering the practical deployment of DKFusion, we explored two modes of human-machine collaboration: (1) Model-driven mode: In this mode, the model aids in diagnosis or decision-making, with humans supervising outcomes. It is ideal for scenarios where high precision minimizes the need for extensive specialist involvement, allowing for rapid evaluations. A common scenario involves routine model usage by specialist. High precision is critical to avoid alert fatigue caused by excessive AI notifications, especially false positives. Field surveys at hospitals highlighted understaffed and overburdened coding departments, making high precision essential as users may not review every predicted missed diagnosis. Another scenario involves batch EMR evaluations, where reliability and precision are vital. In both cases, DKFusion-S is better choice, offering higher precision and minimizing false alerts. Compared to Qwen3-30B-A3B, it achieves higher recall while maintaining comparable precision. (2) Specialist-driven mode: In this mode, human specialists, such as physicians or coders, lead the decision-making process, with the model serving as a supportive tool to reduce workload while ensuring accuracy. A common application is assisting specialists in EMR quality control, where they focus on checking EMR quality, coding diagnoses, and detecting missed diagnoses to create a complete and accurate diagnosis list. Maximizing the model’s F1 score strikes a better balance between reducing specialists’ workload and avoid financial losses. While the Baichuan-M2-32B-SFT model demonstrates high F1 and recall scores, hospital privacy requirements often make API access infeasible, and the substantial computational resources required to deploy a 32B LLM are unaffordable for most hospitals, especially overwhelmed primary care facilities. In contrast, our proposed DKFusion model achieves a similar F1 performance to Baichuan-M2-32B-SFT while offering higher precision and requiring less than 1% of the parameters. This makes DKFusion an effective and practical solution for deployment in hospitals.

Simulated tests using the in-domain test set compared the performance of different modes of human-machine collaboration enabled by our model. The specific performance metrics are provided in Table 5. For the specialist-driven mode, we validated the integration of model recommendations with physician decision-making. This approach led to a 17.1% increase in precision, albeit with a 9.6% reduction in the F1 score compared to doctors directly performing missed diagnosis detection. Additionally, results showed that, ideally, manually reviewing an EMR by physician takes approximately 24 minutes, while using the model for recommendations and expert verification reduces this time to 2.3 minutes per EMR. This reflects a nearly tenfold increase in efficiency, reducing the workload for physicians or coders involved in missed diagnosis detection. For the model-driven mode (DKFusion-S), the system achieves precision comparable to that of physicians while requiring an average of 6.8 seconds to process each EMR, making it well-suited for settings where specialists or coders face heavy workloads.

**Table 5:** Comparison of performance and speed in human-machine modes on the in-domain test set

Methods	Precision	Recall	F1	Infer speed (Time per record)
Doctor	80.8%	77.6%	79.1%	1440.0 seconds
DKFusion followed by doctor (Specialist-Driven)	97.9%	53.9%	69.5%	138.0 seconds
DKFusion-S (Model-Driven)	81.2%	17.1%	28.2%	6.8 seconds

## Discussion

Accurate and comprehensive discharge diagnoses, encompassing both principal and secondary diagnoses, are essential for ensuring high-quality EMRs, enabling effective medical care evaluation, and supporting accurate insurance reimbursement. Despite their importance, predicting missed diagnoses-especially secondary diagnoses-remains a major challenge due to the complex nature of EMRs and sparse representation of diagnostic indicators. Several factors contribute to these challenges: (1) EMR complexity: The voluminous and unstructured nature of EMRs, often containing tens of thousands of tokens, complicates data processing and feature extraction. (2) Sparse indicators for secondary diagnoses: Secondary diagnoses are usually mentioned infrequently and can be scattered throughout the EMR, making it difficult for end-to-end models to capture relevant signals. (3) Large-scale candidate space: Missed diagnoses are not closely tied to the patient’s clinical department, as theoretically, any disease could be identified as missed (though in practice, non-severe and non-urgent conditions are more commonly missed). Therefore, the range of potential candidate missed diagnoses exceeds tens of thousands and demands robust models capable of handling both in-distribution and out-of-distribution cases.

These factors distinguish the missed diagnosis detection task from traditional diagnostic prediction tasks, which typically rely on concise inputs, such as discharge summaries, and involve a limited classification space of a few hundred labels^29–31^. To address above challenges, we propose DKFusion, a data and knowledge cross-level fusion-driven learning framework. DKFusion employs a recall–verify strategy that decomposes the task of predicting numerous diagnoses from lengthy and complex EMRs into more manageable steps, enabling the model to detect potential missed diagnostic evidence from various sections of the EMRs. In parallel, the integration of data and knowledge strengthens the model’s robustness.

From the scenario testing results, it is clear that our proposed model, DKFusion, demonstrates a significant advantage over other conventional methods (expert system, BERT-based method) in terms of precision, recall, and F1 score in both in-domain and out-of-domain testing. Additionally, we evaluated the performance of three advanced LLMs (Llama3.1-8B, Qwen3-30B-A3B, Baichuan-M2-32B) under varying granularities (Section-level vs. EMR-level) and optimization strategies (SFT and multi-agent setups). The results demonstrate that DKFusion outperforms LLMs that rely on standard instruction prompting or general architectural optimizations (e.g., general multi-agent frameworks). Even when LLMs are specifically adapted for the task, DKFusion achieves performance comparable to the best 30B-parameter Baichuan-M2-SFT, while utilizing less than 1% of the parameter count. This underscores DKFusion’s effectiveness and resource efficiency in practical deployment scenarios. Furthermore, these findings highlight the complexity of the missed diagnosis detection task, as even a outstanding 30B-parameter LLM struggles to fully address the challenges in this area. This calls for further research to better tackle the intricacies of missed diagnosis detection.

In this study, we analyzed the impact of missed diagnoses on DRG grouping. Findings from the test set indicate that in the DRG payment system, 9.03% of the cases with missed diagnoses found by our model will result in DRG grouping changes, resulting in an increase of 3.15% in medical insurance payments. This emphasize the important impact of missed discharge diagnoses within the DRG payment system. We also analyzed the current major error cases of false positives and false negatives. While efforts have been made to address these issues, there is still considerable room for optimization. We further investigated two modes of human-machine collaboration, and simulation experiments showed that, with the aid of our models, both modes enhanced specialist’ efficiency in detecting missed diagnoses. Additionally, this method offers interpretability. Unlike end-to-end models that output only a prediction label, the DKFusion approach first identifies potential missed diagnoses and then validates them using contextual evidence from the entire EMR, providing stronger traceability of evidence. This reduces the verification burden on specialist, improves confidence in the model’s outputs, and facilitates better integration into clinical workflows.

Additionally, during our study, we identified several common patterns of missed diagnoses: (1) Omitted imaging findings. Clear abnormalities in imaging studies were not entered into the diagnosis list, for example, pleural effusion and fatty liver, due to physician oversight. (2) Omitted laboratory findings. Abnormal lab results were clinically managed, but not documented as formal diagnoses, for example, hyperlipidemia and hypokalemia. (3) Omitted Complications information. Complications of chronic diagnoses were sometimes left out, for example, hypertensive and diabetic complications. Historically, China’s fee-for-service system treated missed diagnoses as a peripheral record-quality issue, resulting in limited attention. As DRG adoption spreads, we expect growing emphasis on documentation completeness and a corresponding decline in missed-diagnosis rates. Nevertheless, because comprehensive review by physicians and coders remains time-consuming, missed diagnoses are likely to persist as a long-term challenge.

Furthermore, while our experiments were conducted on Chinese EMRs, the proposed DKFusion framework possesses significant potential for generalization to other languages like English and healthcare systems. First, the model architecture is language-agnostic. The core mechanism–fusing data with knowledge–relies on vector representations rather than linguistic rules specific to the Chinese language. Adapting the framework to an English environment primarily involves replacing the text encoder (e.g., using ClinicalBERT^32^ instead of Chinese BERT). Second, the knowledge sources are replaceable with standardized English equivalents. The disease dictionaries utilized in our graph can be aligned with international ontologies such as those in the Unified Medical Language System (UMLS)^33^ or SNOMED CT^34^. The contextual prior knowledge (e.g., NEG) can be mapped to English, while the implicit diagnostic relationship knowledge can be mined and reconstructed from large-scale public English datasets like MIMIC-IV^35^. Finally, the missed diagnosis problem is universal. The logic of DRG payments, which relies on the accurate coding of CC/MCC, is shared by healthcare systems globally (e.g., in the US, Germany, and Australia). Therefore, the utility of our method in identifying high-value missed diagnoses is theoretically transferable across borders. Of course, due to the differences in the structure of EMRs across various languages and healthcare systems, certain adjustments need to be made during actual deployment.

Our study has the following limitations: (1) Although our system theoretically supports missed diagnosis detection for 29,563 ICD-10 coded conditions, it currently demonstrates robust performance on only about 4,274 of those codes, indicating that its effective coverage of diagnoses remains limited. (2) Although we have discussed the theoretical generalizability of our framework to other languages, empirical verification is yet to be conducted. Due to the difficulty of obtaining annotated multilingual data, this study is restricted to Chinese EMRs from multi-regional hospitals, and performance on non-Chinese records has not yet been experimentally verified. (3) Although we have identified the primary causes of prediction errors, our error analysis reveals that many types of long-tail errors remain challenging to define and fully resolve. (4) This approach restricts the scope of detected missed diagnoses to those explicitly documented, excluding conditions inferred solely from clinical signs or symptoms.

In future work, we plan to expand the annotation scope of our test sets to enhance the representativeness of our findings. Our experiments demonstrate the significant potential of LLMs in addressing missed diagnoses, particularly following targeted domain-specific optimization. Leveraging their robust foundational medical knowledge and strong contextual understanding, LLM-based approaches represent a promising pathway toward mitigating diagnostic omission. In our upcoming research, we plan to leverage LLM/agents to explore the following: (1) Expanding the range of covered diagnoses to improve the generality of the model. (2) Incorporating formal diagnostic criteria, rather than solely relying on attending physicians’ descriptions, improves the determination of missed diagnoses. (3) Jointly optimizing the missed diagnosis task with related DRG tasks (e.g., principal diagnosis selection and DRG prediction), since multitask coordination may deliver greater benefits than addressing each task in isolation.

## Methods

**Fig. 6:**
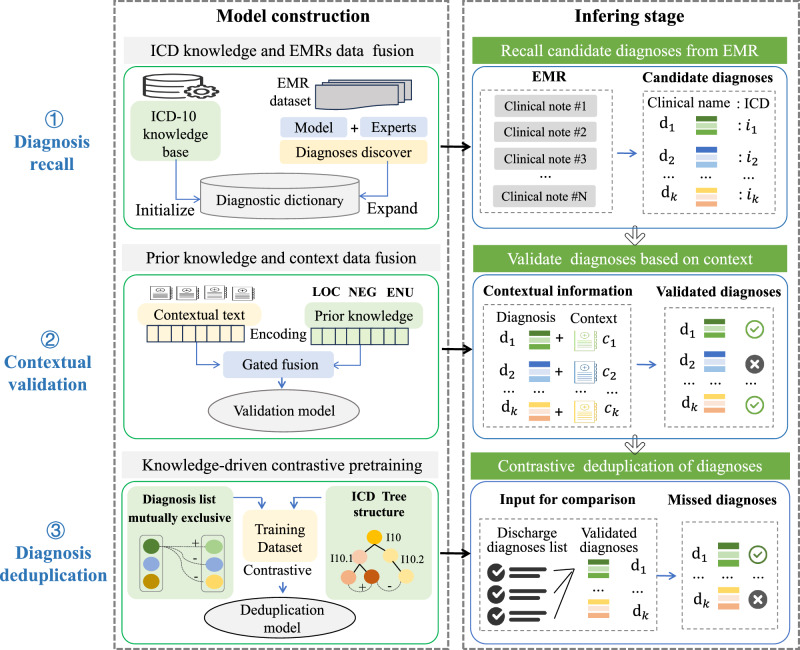
Architecture design of our proposed DKFusion framework. The framework consists of three key steps: retrieving candidate diagnoses from EMR notes using the diagnosis recall module, validating them via the contextual validation module, and deduplicating them against the discharge diagnosis list using the diagnosis deduplication module to identify missed diagnoses. During model construction stage, this framework employs a data-knowledge cross-level fusion approach. Each module incorporates different types of data (represented in light yellow) and knowledge (represented in light green). The process sequentially involves discovering diagnosis knowledge from EMR data, selectively fusing prior knowledge embedding and contextual embedding through gating mechanisms, and generating data from knowledge for contrastive pretraining.

Our DKFusion framework comprises three core modules: diagnosis recall, contextual validation, and diagnosis deduplication. The diagnosis recall module quickly retrieves potential clinical diagnoses from EMRs, the contextual validation module filters out false positives among the retrieved diagnoses, and the diagnosis deduplication module removes redundancies between recalled diagnoses and those already listed in the discharge diagnosis list. The three modules are illustrated in Fig. 6.

All three modules emphasize efficiency, effectiveness, and usability through data-knowledge fusion strategies. Incorporated knowledge spans physician expertise, task-specific priors, EMR diagnosis list structures, and ICD hierarchies. The data includes large-scale EMRs, unsupervised corpora for pretraining, a contrastive diagnostic dataset derived from knowledge, expert-annotated fine-tuning data, and augmented data. Fusion methods include knowledge discovery from EMRs in the recall module, gated integration of prior knowledge and contextual text embeddings in the validation module, and knowledge-to-data generation for contrastive pretraining in the deduplication module.

The three DKFusion modules described above are detailed in Sections “Dictionary-based diagnosis recall,” “Contextual diagnosis validation,” and “Clinically-oriented diagnosis deduplication,” while Section “Training and testing results” details the training and testing procedures. Furthermore, to address scenarios where specialists working under high pressure are prone to alert fatigue caused by frequent false alarms, a precision-prioritized variant, DKFusion-S, is proposed. The implementation details of this model are provided in Section “The implementation of expert system and DKFusion-S.” Sections “Implementation of BERT-based method” and “Implementation of LLM-based method,” respectively, describe the implementation of BERT-based and LLM-based methods.

### Dictionary-based diagnosis recall

As mentioned above, a complete EMR typically contains a substantial volume of text, therefore directly using deep learning-based methods has high complexity during the inference stage. To ensure practical applicability in real-world scenarios, we utilize a diagnostic dictionary-based approach to efficiently recall all potential candidate diagnoses from EMRs. That is

1 $$\{{{\mathcal{D}}}_{EMR},{{\mathcal{V}}}_{diag}\}\to \{{d}_{1},{d}_{2},\cdots \,,{d}_{k}\},$$

where ${{\mathcal{V}}}_{diag}$ is the diagnostic dictionary, *d*_*i*_(*i* = 1, ⋯ , *k*) are candidate diagnoses recalled from a given EMR document ${{\mathcal{D}}}_{EMR}$. The following paragraphs detail the methodology for constructing the diagnostic dictionary and the approach to diagnosis recall and encoding.

**Fig. 7:**
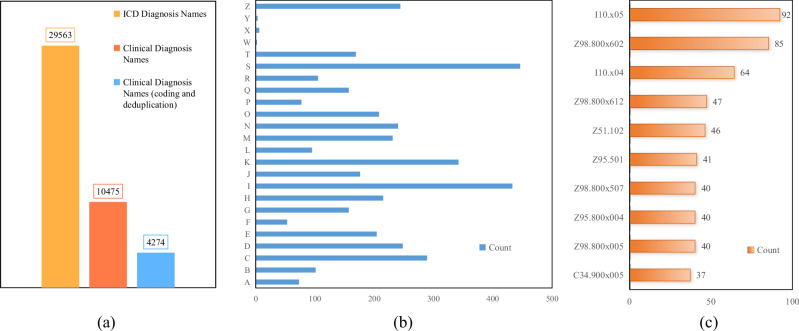
The coverage and distribution of the diagnostic dictionary. **a** Scope of diagnosis support, including: the count of standard diagnosis terms, non-standard clinical diagnosis terms, and unique ICD codes after clinical term mapping and deduplication. **b** The distribution of ICD codes mapped from clinical diagnosis terms, organized by ICD alphabetical prefixes. **c** The top 10 diagnoses with the highest diversity in clinical expression. All mentioned ICD are derived from the publicly available China Medical Insurance ICD-10 version 2.0.

To ensure the suitability of the diagnostic dictionary for clinical diseases, we integrate ICD knowledge with EMR data during its construction, which can be formulated as

2 $$\{{{\mathbb{D}}}_{EMR},{{\mathcal{V}}}_{icd}\}\to {{\mathcal{V}}}_{diag},$$

where ${{\mathbb{D}}}_{EMR}=\{{{\mathcal{D}}}_{EMR}^{1},{{\mathcal{D}}}_{EMR}^{2},\cdots \}$ denotes the set of EMRs used to build the dictionary, ${{\mathcal{V}}}_{icd}$ denotes the ICD knowledge dictionary, and ${{\mathcal{V}}}_{diag}$ denotes the resulting diagnostic dictionary. Specifically, we initially constructed the diagnostic dictionary by collecting 29,563 ICD code-term pairs from the officially published China Medical Insurance ICD-10, comprising standardized diagnostic terms and their corresponding codes. As is well known, ICD-10 demonstrates a significant long-tail effect in real-world scenarios, where a small subset of frequently used ICD codes correspond to numerous variant clinical names. Therefore, expanding the diagnostic dictionary is essential to improve coverage of clinical names encountered in EMRs. In this study, we employed a BERT-BLSTM-CRF model to extract clinical disease names from a large corpus of EMRs, with the results validated by physician experts to ensure accuracy. It should be noted that the BERT-BLSTM-CRF model is used only during the construction of the dictionary and *does not affect the model’s efficiency during the inference stage*. This process ultimately yielded 10,475 clinical diagnosis names, covering the 4274 ICD-10 codes most frequently used in clinical practice. Specifically, infrequently used ICD codes correspond to standardized disease names, whereas frequently used codes are associated with multiple variant clinical names in addition to their standardized designation. The coverage of the diagnostic dictionary and the distribution of diseases are presented in Fig. 7. According to the statistical results shown in Fig. 7(c), there is considerable variation in diagnostic clinical names, with the top 10 ICD codes each associated with between 37 and 92 different clinical names.

After obtaining the diagnostic dictionary ${{\mathcal{V}}}_{diag}$, candidate diagnoses {*d*_1_, *d*_2_, ⋯ , *d*_*k*_} can be efficiently recalled from the given EMR document ${{\mathcal{D}}}_{EMR}$ based on the dictionary-text matching schema, as shown in Eq. (1). Evaluation on a real-world EMR dataset showed that, with sufficient coverage of diagnostic clinical names, the dictionary-based method achieved a precision of 0.705 and a recall of 0.803, compared with 0.895 and 0.649, respectively, obtained by directly applying the sequence labeling model BERT-BLSTM-CRF during the inference stage (further details are provided in Supplementary Table 1), while preserving ease of deployment within existing clinical informatics systems. The precision of disease recall will be further improved during the diagnosis validation stage. Furthermore, since the constructed diagnostic dictionary inherently contains the mapping between clinical disease names and their corresponding ICD codes, candidate diagnoses can be rapidly assigned ICD codes through this mapping without requiring an additional ICD coding model:

3 $$\{\{{d}_{1},{d}_{2},\cdots \,,{d}_{k}\},{{\mathcal{V}}}_{diag}\}\to \{\{{d}_{1}:ic{d}_{1}\},\{{d}_{2}:ic{d}_{2}\},\cdots \,,\{{d}_{k}:ic{d}_{k}\}\},$$

where *icd*_*j*_ (*j* = 1, ⋯ , *k*) denotes the ICD-10 code of the recalled diagnosis *d*_*j*_. Therefore, we leveraged it for efficient and practical ICD coding in the DRG impact analysis experiment (Section Error analysis). A brief review of miscoding revealed only two cases (out of 27 cases correctly grouped) that impacted the final DRG, indicating that dictionary-based ICD coding is cost-effective and relatively reliable under the DRG system; details of these edge cases are in Supplementary Table 7.

### Contextual diagnosis validation

Candidate diagnoses recalled from EMRs require subsequent validation to determine their relevance to the current patient and thereby reduce potential false positives. Such clinical disease names do not necessarily reflect the patient’s actual condition; they may appear in a physician’s differential diagnosis, within contextual discussions of related medical knowledge, or in other non-diagnostic contexts. Diagnosis validation primarily relies on the contextual information surrounding a disease name in the EMR, including both the semantic content of nearby clinical text and contextually relevant domain-specific prior knowledge. Take the candidate diagnosis *d* as an example, the diagnosis validation process can be formalized as:

4 $$\{d,c,{{\mathcal{K}}}_{{\rm{prior}}}\}\to {y}_{valid},$$

where *c* represents the contextual clinical text, ${{\mathcal{K}}}_{{\rm{prior}}}$ denotes the prior knowledge, and the output *y*_*valid*_ ∈ {yes, no, uncertain} indicates *whether the diagnosis is confirmed, unconfirmed, or uncertain*. The following briefly introduces the encoding of contextual clinical text, the encoding of prior knowledge, and the integration of textual semantics with prior knowledge for validation decision-making.

In this study, we employed a pre-trained BERT model to encode the semantic features of the contextual clinical text:

5 $${h}_{c}=Relu({W}_{c}^{{\rm{T}}}({F}_{bert}(concat(d;c)))+{b}_{c}),$$

where concat (*d*; *c*) denotes the character-level concatenation of the diagnosis term *d* and its contextual clinical text *c*; *F*_*bert*_(⋅) represents the text encoding performed by BERT; the *Relu*(⋅) denotes the ReLU activation function; *W*_*c*_ is a learnable weight matrix of dimension ${{\mathbb{R}}}^{768\times 768}$; and *b*_*c*_ represents the bias parameters. It should be noted that the diagnosis *d* is concatenated at the front of the context to serve as a clear indicator, distinguishing it from other possible diagnoses included within the context.

To enhance the effectiveness of diagnosis validation, this study integrates three types of contextual prior knowledge encoding: (1) diagnosis location in context (LOC) to reduce distraction from other diagnoses; (2) negation (NEG) to interpret negative meanings^36^; and (3) enumerative diagnostic structures (ENU), indicating that all diagnostic items share the same confirmation status. These types of prior knowledge can be represented through the state of each token within the contextual window. For example, the position of the disease term is marked as “1” while all other positions are marked as “0”; similarly, tokens indicating negation are marked as “1” and non-negation tokens as “0”; tokens within enumerations of diseases are marked as “1” and those outside as “0”. Supplementary Fig. 2 details a case of prior knowledge feature processing. The different states of the three types of prior knowledge are represented using distinct trainable embeddings; thus, the corresponding contextual prior knowledge is encoded via an embedding lookup operation:

6 $${{\bf{E}}}^{loc}[{f}_{loc}],\,{{\bf{E}}}^{neg}[{f}_{neg}],\,{{\bf{E}}}^{enu}[{f}_{enu}]\to \{em{b}_{loc},\,em{b}_{neg},\,em{b}_{enu}\}$$

where *f*_*loc*_, *f*_*neg*_, and *f*_*enu*_ denote the prior knowledge state inputs for LOC, NEG, and ENU respectively, with respect to the context *c*, **E**^*loc*^, **E**^*neg*^ and **E**^*enu*^ are the corresponding embedding lookup tables, from which *emb*_*loc*_, *emb*_*neg*_, and *emb*_*enu*_ are obtained as the respective prior knowledge feature embeddings. The prior knowledge feature embeddings are further integrated through a neural network layer:

7 $${h}_{k}=Relu({W}_{loc}^{{\rm{T}}}em{b}_{loc}+{W}_{neg}^{{\rm{T}}}em{b}_{neg}+{W}_{enu}^{{\rm{T}}}em{b}_{enu}+{b}_{k}),$$

where *W*_*loc*_, *W*_*neg*_ and *W*_*enu*_ are trainable parameter matrices of dimension ${{\mathbb{R}}}^{200\times 200}$, and b_*k*_ denotes the bias term.

After obtaining the contextual text embedding *h*_*c*_ and the fused prior knowledge embedding *h*_*k*_, the two feature representations are integrated using a gated fusion mechanism:

8 $$o=g\odot {h}_{f}+(1-g)\odot {h}_{c},$$

where the gating vector $g=sigmoid({W}_{g}^{{\rm{T}}}[{h}_{c};{h}_{k}]+{b}_{g})$ controls the relative contribution of the fused features, and ${h}_{f}=tanh({W}_{f}^{{\rm{T}}}[{h}_{c};{h}_{k}]+{b}_{f})$ denotes the coarse fusion of textual and prior knowledge features. Subsequently, token-level embeddings are aggregated into fixed-length summary vectors using both max-pooling and mean-pooling operations. The concatenated pooled representation is passed through a softmax classification layer to assign probabilities to three diagnostic states-confirmed, unconfirmed, and uncertain-by optimizing the cross-entropy loss function:

9 $$y=softmax({W}_{p}^{{\rm{T}}}[maxpool(o);meanpool(o)]+{b}_{p}),$$

where *W*_*f*_, *W*_*g*_, *W*_*p*_, *b*_*f*_, *b*_*g*_ and *b*_*p*_ are trainable parameters. In modules training and testing phase, to address data limitations and improve robustness, random word and diagnosis replacement data augmentation was used to expand the dataset by 50,000 samples.

Ablation studies and comparison experiments with baselines demonstrate that, our model outperforms the baseline mainstream models like BERT-based method and LLM-based method in contextual validation task. All key features contributed, with LOC having the most significant impact (F1 improved by 2.5%), while NEG had less effect (F1 improved by 0.2%) due to the limited number of negative terms, which were likely already well-learned from the training data. The above experimental results, as shown in Supplementary Table 4, validate the effectiveness of our proposed gate-mechanism-based knowledge and data fusion method. For the discussion on non-confirmed cases and the case study, please refer to Supplementary Fig. 1 and Supplementary Fig. 4, respectively.

### Clinically-oriented diagnosis deduplication

The above validation process confirms whether diagnoses recalled from EMRs are truly patient conditions. In real-world clinical deployment, physicians expressed resistance when the AI system duplicated diagnoses already included in the initial discharge list, emphasizing the need to precisely identify and supplement missed diagnoses without redundancy. To address this, a robust deduplication process is required to reconcile validated candidate diagnoses with the initial discharge diagnosis list. However, such deduplication is highly challenging and goes beyond simple string matching, as clinicians often use different terminologies for the same condition-for example, “pulmonary inflammatory changes” in imaging reports versus “pulmonary infection” in discharge diagnoses. Therefore, the diagnosis deduplication module must not only identify semantic relationships across tens of thousands of diagnoses but also account for the variability in how conditions are expressed by clinicians in practice.

The clinically-oriented diagnosis deduplication module addresses this challenge by evaluating relationship between validated diagnosis and each discharge diagnosis. This process can be formalized as:

10 $$\{{d}_{validated},{d}_{discharge}\}\to {y}_{dedup},$$

where *d*_*validated*_ and *d*_*discharge*_ represent the validated candidate diagnosis and the discharge diagnosis, respectively. The output *y*_*dedup*_ indicates relation of *d*_*confirmed*_ and *d*_*discharge*_ is similar, included, secondary, or irrelevant. The four clinically-oriented diagnostic relationships are categorized as follows: (1) Similar, where candidate and discharge diagnoses describe overlapping or highly related medical conditions, making them interchangeable and marked as duplicates; (2) Inclusion, where one diagnosis is a subset or broader category of the other, indicating a hierarchical relationship, often describing the same condition within an EMR and is therefore marked as duplicates; (3) Secondary, where diagnoses are indirectly connected, such as through complications or consequences, but are not considered duplicates; and (4) Irrelevance, where diagnoses are entirely unrelated and lack any meaningful medical connection, also not considered duplicates. These relationships are bidirectional, meaning the classification applies regardless of whether the candidate diagnosis includes or is included by the discharge diagnosis. Further examples of these categories are provided in Supplementary Table 2.

Determining the relationships between diagnoses can be viewed as a short-text classification problem. The challenge lies in the high information density of diagnostic text and its strong domain-specific nature. Encoding through standard methods, such as pretrained models trained on general unsupervised corpora, often fails to effectively capture clinically-oriented diagnostic semantic relationships due to a lack of sufficient diagnosis relationship knowledge. To address this, we infused diagnostic relationship knowledge to pretrained model using contrastive learning. The following section introduces the knowledge-driven dataset construction and training methods.

To effectively construct a contrastive dataset, we leveraged two types of diagnostic relationship knowledge to generate a large number of training pairs: (1) The mutual exclusivity of diagnoses in the diagnosis list: We utilized historical EMR datasets containing documented mutual exclusivity discharge diagnoses and their corresponding ICD-10 codes as knowledge-information systematically documented on patient face sheets by coders. Diagnoses with different clinical expressions that matched their corresponding ICD-10 standard names were included as positive pairs, while mismatches between different diagnoses were considered as negative pairs. (2) ICD tree structure: we leveraged the hierarchical structure of the China’s medical insurance ICD-10 (v2.0) coding system as knowledge. Such as diagnoses sharing identical ICD-10 four-digit codes were regarded as positive pairs, whereas diagnoses falling under distinct or distant ICD-10 codes were classified as negative pairs.

Next, contrastive pretraining is required for diagnosis deduplication module to infuse diagnostic relationship knowledge. By leveraging the data generated by the above two types of diagnostic relationship knowledge and combining it with common data augmentation techniques such as back-translation, we generated a task-specific contrastive dataset containing 302,000 positive pairs and 410,000 negative pairs. We utilized the BERT model as the pretrained model and applied the following SimCSE contrastive loss^37^ during training on this dataset to inject diagnostic relationship knowledge:

11 $$Los{s}^{Contrastive}=-\log \frac{{e}^{sim({h}_{i},{h}_{i}^{+})/\tau }}{{\sum }_{j=1}^{N}({e}^{sim({h}_{i},{h}_{j}^{+})/\tau }+{e}^{sim({h}_{i},{h}_{j}^{-})/\tau })},$$

where *N* represents the mini-batch size, *h*_*i*_ = *F*_*BERT*_(*d*) denote the diagnosis embedding encoded by BERT model, ${h}_{j}^{+}$ denotes embedding of positive samples, ${h}_{j}^{-}$ represents embedding of negative samples, *τ* is the temperature parameter, and *sim*(*h*_1_, *h*_2_) function represents cosine similarity $\,{\rm{sim}}\,({h}_{1},{h}_{2})=\frac{{h}_{1}\cdot {h}_{2}}{\parallel {h}_{1}\parallel \cdot \parallel {h}_{2}\parallel }$ between *h*_1_ and *h*_2_. During training, each mini-batch is constructed with samples $({d}_{i},{d}_{i}^{+},{d}_{i}^{-})$, representing a diagnosis, a positive example, and a negative example, respectively. These are randomly sampled from the aforementioned contrastive dataset. By leveraging these two types of knowledge, encompassing different clinical expressions and extensive diagnostic relationship knowledge, contrastive learning effectively enhances the pretrained model’s diagnostic discrimination capabilities, addressing the challenge of clinically-oriented diagnosis deduplication.

Subsequently, to enable the model to acquire specific diagnostic relationship judgment capabilities, the module was fine-tuned using supervised training with expert-annotated diagnostic pairs, categorized into the four aforementioned diagnostic relationship classes. A four-class cross-entropy loss was employed during this supervised fine-tuning process to enable the model to achieve fine-grained classification of relationships between diagnoses.

In modules training and testing phase, the ablation results and comparison experiments with baselines demonstrate that our proposed model outperformed traditional deep learning baselines and LLM-based few-shot methods. Our contrastive data construction method improves the performance of the pre-training model. Notably, the method based on diagnostic exclusivity in diagnosis list has the greatest impact (F1 improved by 2.2%), while common text data augmentation methods offer minimal improvement (F1 improved by 0.5%). The ablation results confirm that, using the same fine-tuning data, our method effectively provides the pre-training model with additional expert knowledge, thereby improving its performance. More details are provided in Supplementary Table 5. For the case study related to this module, please refer to Supplementary Table 6.

### Training and testing details

This section details the modules training and testing phase, the scenario testing phase, and the approach used to handle lengthy EMRs.

By randomly splitting the data into an 8:2 ratio for the training and testing sets, we evaluated the performance of the contextual validation and diagnosis deduplication modules. In the training set, we reserved 20% of the data as a validation set for model selection. For the diagnosis recall module, as it uses a dictionary-based approach, its performance was directly evaluated on the test set. In training process, all lookup tables **E** are initialized using the Xavier strategy^38^ and updated during training, while all *W* weight parameters are initialized using the Kaiming initialization method^39^. The learning rate for the two submodules (contextual validation and diagnosis deduplication) is set to 5e-5, with a maximum training duration of 10 epochs. Early stopping is employed for optimal model selection, where the training process is halted if the validation loss does not improve for 20 consecutive training steps. The value of *τ* in contrastive learning is set to 0.05. More detailed training information and hyperparameters are provided in Supplementary Table 3.

For scenario-based in-domain and out-of-domain testing, we selected the patient face sheet as the source for the discharge diagnosis list. The face sheet, completed by the attending physician following the discharge summary and subsequently verified by medical coders, contains the principal and secondary diagnoses together with their associated ICD codes. We combined the principal and secondary diagnoses to create the final discharge diagnosis list. To ensure result stability, each model was executed three times during testing, with the average performance reported. During the evaluation, DKFusion employed a standard confidence threshold of 0.5 for both the contextual validation and diagnosis deduplication modules. In a probabilistic framework, a threshold of 0.5 provides a natural baseline for an “absolute majority," guaranteeing a high level of confidence in default predictions without test-set tuning. To assess threshold robustness, we conducted a sensitivity analysis via grid search (Supplementary Fig. 10). The results indicate relatively stable model performance across moderate thresholds (validation module: 0.33–0.7; deduplication module: 0.25–0.5), whereas high thresholds (e.g., 0.9) degrade performance by either over-filtering valid diagnoses or overlooking actual missed diagnoses. Thus, although the 0.5 threshold does not achieve the optimal F1 performance (61.2% vs. 63.8%), it provides an effective balance between precision and recall for final deployment.

EMRs are often lengthy (with an average length of 20,437 tokens in the test set), making them challenging for models to process. The DKFusion model’s recall-verify pipeline effectively mitigates this issue: the diagnosis recall module, based on dictionary, excels at identifying relevant information in long texts, while the subsequent two modules focus only on localized contexts to avoid directly processing the entire EMR. For the context validation module, constrained by its textual encoder to a maximum length of 512 tokens, addresses this limitation by splitting the relevant notes into sentences and extracting the recalled diagnosis sentence along with its immediate predecessor and successor. The input is formatted as “[CLS] diagnosis [SEP] context” and truncated from the right to fit within the 512-token limit. Fewer than 2.4% of cases exceed this length, meaning critical information is rarely lost (see Supplementary Fig. 3). For the diagnosis deduplication module, the input is formatted as “[CLS] confirmed diagnosis [SEP] discharge diagnosis”. Since diagnosis names are relatively short (fewer than 30 tokens), no special handling of input length is required.

### The implementation of expert system and DKFusion-S

In real-world deployment scenarios with high clinical pressure, physicians or coders may lack the capacity to thoroughly verify the model’s recommendations. To prevent frequent errors that cause alert fatigue in such scenarios, we designed DKFusion-S to prioritize precision. DKFusion-S adopts a hybrid approach, combining DKFusion with expert systems. The following paragraphs primarily describe the implementation of the expert system, how the expert system is integrated with the DKFusion method, and the role of the expert system in DKFusion-S.

The expert system follows the same workflow as DKFusion during implementation. They share the same diagnosis recall module, but the expert system’s subsequent two modules are implemented using rule-based methods, as detailed below: (1) The contextual validation module operates on two levels. At the paragraph level, if terms such as “differential diagnosis” or “informed consent” appear, any diagnoses mentioned thereafter within the same paragraph are classified as unconfirmed. At the sentence level, candidate sentences are checked for negation words, and any match leads to invalidation of the corresponding diagnosis. (2) The diagnosis deduplication process also incorporates two steps. First, ICD code comparison is performed, where two diagnoses are considered similar if their ICD codes share the same first four digits, and different otherwise. Second, a similarity score is calculated using the edit distance method and a threshold-based exclusion mechanism is applied to eliminate redundant predictions corresponding to discharge diagnoses.

The proposed expert system and DKFusion model both adhere to the recall-and-verify framework, allowing them to be combined modularly, as detailed below: (1) Context validation module: If either the expert system or the model indicates that a diagnosis is unconfirmed or uncertain, it is excluded from further consideration. (2) Diagnosis deduplication module: If either the expert system or the model suggests that a diagnosis is duplicated with the discharge diagnoses, it will be removed from the candidate missed diagnoses.

The expert system plays an important role in enhancing the precision of the DKFusion-S model. By providing stable judgments for straightforward cases, it reduces false positives more effectively than threshold adjustments alone, lifting out-of-domain precision to 83.5% compared to the 78.0% achieved with 0.95 confidence thresholds across two modules. While DKFusion, as a probabilistic model, which may retain marginal uncertainty even with clear negation markers, the expert system offers more definitive outcomes. However, the expert system’s rigid decision-making process in complex cases results in a higher rate of false negatives, highlighting its limitations in handling nuanced scenarios.

### Implementation of BERT-based method

As a milestone in deep learning, BERT is widely utilized in natural language processing tasks^40^. We used the same pipeline and annotated training data as DKFusion but with different contextual validation and diagnosis deduplication module implementations, employing BERT-based method as a comparative baseline to evaluate the advantages of our approach. A more detailed description of the BERT-based method and its data usage is provided in Supplementary Table 8.

### Implementation of LLM-based methods

With the rapid evolution of LLMs, assessing their potential in missed diagnosis detection has become increasingly essential. While proprietary models like GPT-4^41^ exhibit strong generalization capabilities, strict data privacy protocols and third-party API restrictions in healthcare settings have necessitated the exploration of deployable open-source alternatives. To ensure a comprehensive and fair comparison, we expanded our evaluation beyond basic zero-shot prompting by incorporating different types of LLMs, SFT, and medical multi-agent architectures.

We selected three representative models: 1) Llama-3.1-8B^19^, representing general-purpose capabilities in smaller-scale models; 2) Qwen3-30B-A3B^20^, incorporating a Mixture-of-Experts (MoE) architecture with enhanced reasoning capabilities using “thinking” mode; and 3) Baichuan-M2-32B^21^, a leading medical-specific LLM that consistently outperforms other open-source models on benchmarks such as HealthBench^42^.

We implemented three distinct evaluation strategies to comprehensively test the performance of LLMs in the task of missed diagnosis detection. (1) Section-level vs. EMR-level Inference: To investigate the impact of EMR context length, we evaluated models in two modes: the section-level approach, which processes clinical sections individually and merges the predicted missed diagnoses, versus the EMR-level approach, which processes the comprehensive EMR–including all key clinical documents such as admission, progress, and discharge notes–as a single input. (2) SFT: Supervised fine-tuning is often used as a common optimization method for LLMs, enabling effective alignment of their reasoning capabilities on specific tasks and improving task performance through the use of a small amount of high-quality chain-of-thought (CoT) data^43,44^. To establish a stronger baseline for fine-tuned LLMs, we further fine-tuned the best-performing base model (Baichuan-M2-32B). However, adapting LLMs for missed diagnosis detection presents special data challenges. Two direct methods for constructing training data showed significant flaws in preliminary experiments: 1) DKFusion’s module-level training data cannot be directly transferred to EMR/section-level missed diagnoses; 2) Using raw discharge diagnosis lists to construct EMR-level missed diagnoses introduces distribution noise and modality mismatch, leading to a decline in performance in preliminary tests. Instead of raw-data training, we used a “Model-in-the-loop” approach to curate a high-quality CoT training set from the complete HB dataset (excluding test samples) to enhance task reasoning. Specifically, we collected inference outputs–including both reasoning traces and predicted missed diagnoses from Baichuan-M2. Medical experts then reviewed and corrected these samples to align the task definition, reasoning process, and diagnostic conclusions. This process yielded 992 instruction-alignment training set (covering both EMR-level and section-level scenarios). The input prompt includes task definition and requirements, EMR text, and discharge diagnoses. Detailed prompt templates are provided in Supplementary Fig. 6. During SFT training, we set the learning rate to 1e-5 and trained for 3 epochs. (3) Agent-based Framework: We adopted MDAgents^22^, a leading general-purpose agentic framework for medicine, using Baichuan-M2 as the backbone to simulate multi-turn clinical reasoning. During inference, using a prompt identical to Supplementary Fig. 6, MDAgents autonomously decided to recruit different experts for discussion, aggregating answers and outputting predictions of missed diagnoses. All LLM-related experiments were conducted with the settings of temperature=0.6 and top_p=0.95.

#### Ethics

This study was approved by the Institutional Review Board of Beijing University of Posts and Telecommunications (Approval No. BUPT-P-2025011). All the clinical data was totally de-identified to ensure confidentiality and privacy.

## Supplementary information

Supplementary information

## Data Availability

The datasets used and analyzed during the current study are available from the corresponding author on reasonable request.

## Code availability

The code for DKFusion and DKFusion-S in this study is available from the corresponding authors upon reasonable request.
