# Supplementary material for: A data and knowledge cross-level fusion-driven learning framework for detecting missing diagnosis
Source: NPJ Digit Med. 2026 May 14;9:580. doi: 10.1038/s41746-026-02725-z (PMC13408341; doi:10.1038/s41746-026-02725-z)
Supplement: Supplementary file 1 — Supplementary information [file 41746_2026_2725_MOESM1_ESM.pdf]

Supplementary Information

1 Addition Details of the Module

1.1 Comparison of diagnosis-recall performance

To construct the diagnostic dictionary for the diagnosis recall module, we first trained a BERT-BLSTM-CRF sequence model to identify diagnoses, which serves as the tool for dictionary construction. The training data were sourced from the CCKS-2019 challenge, which offers a collection of 1,000 annotated medical datasets suitable for training and evaluating medical NER models.

Subsequently, based on this sequence model, we constructed the diagnostic dictionary using the following steps: 1) Considering department coverage and ensuring diversity in principle diagnoses, we randomly selected 10,000 EMRs and segmented them into individual sentences. 2) We utilized the sequence model to identify candidate diagnostic terms that appeared at least twice. 3) Physicians then annotated these terms to confirm their validity as diagnostic names and assigned corresponding ICD-10 codes with the help of coding tools. The validated diagnostic names and ICD codes were subsequently compiled to diagnostic dictionary.

Supplementary Table 1 Experiment result of diagnosis recall module. P, R, and F represent precision, recall, and F1-score, respectively.

model	CCKS2019-test			Our annotation testset			Overall		
	P	R	F	P	R	F	P	R	F
BERT-BLSTM-CRF	0.924	0.764	0.836	0.865	0.533	0.660	0.895	0.649	0.748
Dictionary-base (Ours)	0.608	0.839	0.705	0.802	0.767	0.784	0.705	0.803	0.745

As shown in Supplementary Table 1, we evaluated both the BERT-BLSTM-CRF model and our dictionary-based method in two sets of tests: (1) the official CCKS-2019 benchmark test set (379 cases with 1,323 diagnoses) and (2) our custom diagnosis recall test set consisting of 103 randomly sampled EMRs sentences containing 490 manually annotated diagnoses. The data used to construct the dictionary has no overlap with the source of this test set.

The evaluation reveals critical differences between the BERT-BLSTM-CRF and Dictionary-base methods: For BERT-BLSTM-CRF, the CCKS2019-Test set represents in-distribution data (sharing training data origins), while for dictionary-base method

it constitutes out-of-distribution data. This distinction, combined with annotation discrepancies (our labeled diagnoses included conditions unmarked in CCKS2019-Test), led to Dictionary-base's lower precision on this benchmark. Nevertheless, Dictionary-base achieved higher recall (0.839) than BERT-BLSTM-CRF (0.764). More notably, on our custom test set, BERT-BLSTM-CRF performance dropped substantially (F1:0.660), while Dictionary-base maintained strong performance (F1:0.784). These findings show that, when built with sufficient coverage, dictionary-based methods can still deliver reasonable performance on out-of-distribution datasets. In contrast, deep-learning–based approaches require large amounts of training data and experience a decline in generalization when data are limited. The false positive errors identified by the dictionary method are primarily due to: (1) Annotation discrepancies, where the understanding of diagnoses differs between the physicians annotating the test set and those validating the dictionary. (2) A small number of conditions are determined as diseases based on contextual variations.

1.2 Contextual validation

Diagnosis	Context	Type of non-confirmed
Diabetes	...Denial of **Diabetes** and Hypertension...	Negative Diagnosis
Leukemia	...It is necessary to differentiate from diseases such as **leukemia** and agranulocytosis...	Differential Diagnosis
Pelvic Inflammatory Disease	...**Pelvic inflammatory disease** is a common condition among women...	Diagnosis Knowledge Introduction
Coronary Heart Disease	...Currently, the possibility of the patient having **coronary heart disease** cannot be ruled out....	Uncertain Diagnosis

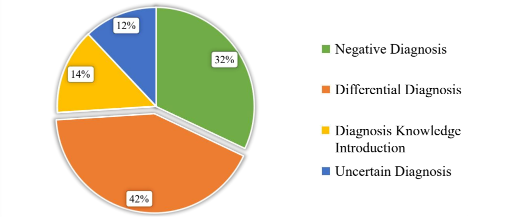

Supplementary Figure 1 Analysis of diagnoses in non-confirmed cases. The ratios depicted in the figure are derived from our statistical analysis of various situations present in actual EMRs.

Due to the complexity of the EMRs, when doctors describe a diagnosis in the EMR, it is not necessarily a confirmed diagnosis. A fter our specific analysis, there are mainly four types of non-confirmed situations, and the specific situations are shown in Supplementary Figure 1. We adopt the contextual validation module for each recalled diagnosis to classify whether it is a confirmed diagnosis by analyzing the context. The following are some additional details about this module.

The processing of prior knowledge features. The features we use include the following: (1) The location of diagnosis in context (LOC). Using regular expressions to

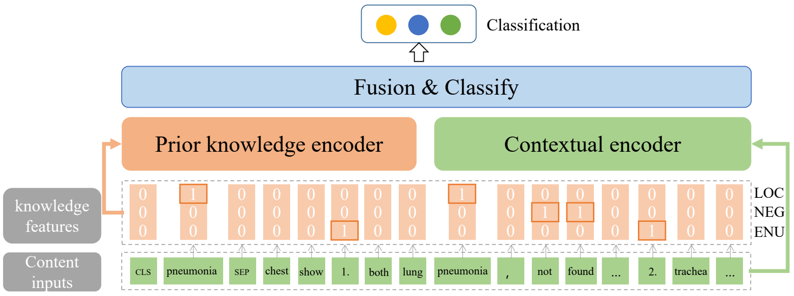

Supplementary Figure 2 A case of prior knowledge feature processing in the contextual validation module. The input of the model is diagnosis and its context, and through the prior knowledge encoder and contextual encoder. Finally, the relationship classification between diagnosis and context is obtained through fusion and classification layers. LOC, NEG, and ENU respectively represent the diagnosis location feature, negation words feature, and the enumerative diagnostic expressions structures feature.

match the diagnosis directly in the context to determine the position of the diagnosis in the context. Then, set the location where the diagnosis occurs to 1 and other locations to 0. (2) The negation words in context (NEG). We first collect the Chinese medical negative word list involved physicians and then use it in the context. In fact, we collected 23 of the most common negation words. If there are words in the negative word list, they are marked as 1, otherwise, they are 0. (3) The enumerative diagnostic expressions structures in context (ENU). Since the presence of sequence numbers often indicates enumerative grammar, we assign '1' to sequence number positions and '0' to all other positions. An example of prior knowledge feature processing in the contextual validation module is shown in Figure 2.

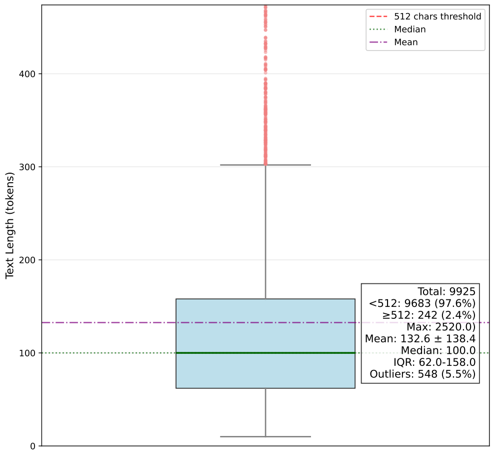

Supplementary Figure 3 Distribution of token lengths. Token lengths of concatenating confirmed diagnoses with their clinical contexts (n=9925 instances).

Input length analysis. Due to the 512-token input limitation of the textual encoder (implemented with BERT), we systematically evaluated potential information loss in our framework. By analyzing real-world annotated data distributions, we found that only 2.4% of disease-context concatenations exceeded the token limit, as demonstrated in Supplementary Figure 3.

1.3 Diagnosis deduplication

After ensuring that the recalled diagnoses are confirmed, it is necessary to exclude those that have already been correctly recorded in the discharge diagnosis list. These different recording scenarios encountered in clinical practice make it highly challenging to determine whether a confirmed diagnosis is duplicated with a discharge diagnoses. We defined four different diagnostic relationships and examples of these four types can be found in Supplementary Table 2.

Supplementary Table 2 Examples of comparison between recalled and recorded diagnoses with corresponding types.

Candidate diagnosis	Discharge diagnosis	Relation	Duplication
Pneumonia	Pulmonary Infection	Similar	Y
Fracture of the Left Hand	Upper Limb Fracture	Inclusion	Y
Bronchitis	Chronic Obstructive Pulmonary Disease (COPD)	Secondary	N
Diabetes	Thoracic Malignant Tumor	Irrelevance	N

Considering the input space complexity, the total number of diagnoses, based on the ICD-10 standard, exceeds 30,000 in the Chinese insurance version v2.0, leading to 900 million possible diagnostic input combinations. However, with only 4,999 annotated entries, specialized techniques are necessary to gather comprehensive information for diagnosis comparison. To address this, we developed a data augmentation technique to collect diagnostic relationship knowledge, enabling the model to learn these relationships through contrastive pre-training. This is followed by fine-tuning on annotated data to train a diagnostic comparison model that meets the requirements.

The detailed process for constructing the contrastive dataset. The process is: (1) The mutual exclusivity of diagnoses in the diagnosis list: We collected medical records and extracted discharge diagnosis lists structured as "code 1: clinical diagnosis 1; code 2: clinical diagnosis 2". ICD-10 codes were mapped to standard diagnosis names, creating positive examples such as (standard diagnosis name 1, clinical diagnosis name 1). Negative examples were created using other combinations. (2) ICD tree structure: Negative examples were constructed by pairing standard diagnosis names from related ICD codes. Diagnoses with the same four-digit code were considered semantically similar (positive examples, e.g., S30.301 and S30.302), while those with different four-digit codes were considered semantically different (negative examples, e.g., S30.301 and S30.401). (3) common data augmentation: Diagnoses from the

training set constructed by the first two methods were augmented using back translation, where one diagnosis was translated into another language and then back into the original language, forming new pairs with the same labels as the original pairs. Using these methods, augmented data were constructed for the diagnosis comparison module, consisting of 302,000 similar disease pairs and 410,000 dissimilar disease pairs.

2 Modules Training and Testing Results

For the experiments of the sub-modules, we used their respective annotated data and randomly divided the training set and the testing set according to the ratio of 8:2. We used 4 NVIDIA GeForce RTX 3090 graphics cards for experiments.

Some key parameters set as Supplementary Table 3. We use accuracy, macro precision, macro recall, and macro F1 for evaluation. For the calculation method of macro precision, macro recall, and macro F1, just calculate the precision, recall, and F1 of each category and then average them.

Supplementary Table 3 Hyperparameter settings

hyperparameter	Contextual validation	Diagnosis deduplication
Epoch	10	10
Batch size	64	200
Learning rate	5e-5	5e-5
Pre-trained model	BERT-base	BERT-base
Max length	512	60
Loss	cross entropy	cross entropy
τ for contrastive loss	-	0.05
Number of categories	3	4

2.1 Contextual validation

Supplementary Table 4 Experiment Result & ablation experiment of contextual validation. The indicators P, R, and F represent the macro average of precision, recall, and F1.

Model	Accuracy	P	R	F
LSTM	0.811	0.778	0.741	0.760
BERT-based	0.898	0.863	0.824	0.841
GPT3.5-turbo	0.805	0.531	0.567	0.548
Llama3-8B-instruct	0.796	0.564	0.588	0.569
Llama3-8B-instruct-SFT	0.886	0.774	0.734	0.751
**Ours**	**0.918**	**0.895**	**0.869**	**0.880**
w/o word replace augment	0.914	0.889	0.867	0.877
w/o diagnosis replace augment	0.913	0.891	0.856	0.872
w/o NEG feature	0.911	0.874	0.867	0.870
w/o ENU feature	0.908	0.870	0.861	0.866
w/o LOC feature	0.898	0.863	0.824	0.841

As shown in Supplementary Table 4, our model outperforms the baseline mainstream models in contextual validation task, achieving the best results. Two leading LLMs, GPT3.5-Turbo and Llama3-8B-instruct, were tested using a 6-shot approach, achieving accuracies of 80.5% and 79.6%, respectively. After supervised fine-tuning, Llama3-8B-instruct-SFT improved accuracy by 9.0% and F1 by 18.2%, but still underperformed compared to BERT-based models and ours. Case analysis revealed similar performance to BERT in confirmed and unconfirmed categories (F1 score difference <2%), but significantly weaker performance in uncertain (F1: 51.1%, over 20% lower than other models), likely due to LLMs' reliance on language understanding, limiting their ability to handle ambiguous categories.

As revealed in the ablation study of our model shown in the Supplementary Table 4, performance decreases when data augmentation and prior features are removed, eventually aligning with the basic BERT-based model. All key features contributed, with LOC having the most significant impact (2.5%), while NEG had less effect (0.2%) due to the limited number of negative terms, which were likely already well-learned from the training data. The above experimental results validate the effectiveness of our proposed gate-mechanism-based knowledge and data fusion method.

2.2 Diagnosis deduplication

The diagnosis deduplication task can be considered a medical short text matching task. We selected LSTM and BiMPM [1] as representatives of Siamese and interaction-based models, BERT as a representative of Masked language models, and GPT3.5-turbo, Llama3-8B-instruct and Llama3-8B-instruct-SFT as representatives of generative language models.

Supplementary Table 5 Experiment result & ablation experiment of diagnosis deduplication. The indicators P, R, and F represent the macro average of precision, recall, and F1.

model	Accuracy	P	R	F
LSTM	0.685	0.651	0.638	0.641
BIMPM	0.721	0.713	0.678	0.695
BERT-based	0.821	0.770	0.762	0.764
GPT3.5-turbo	0.629	0.572	0.565	0.545
Llama3-8B-instruct	0.515	0.513	0.523	0.482
Llama3-8B-instruct-SFT	**0.882**	0.820	**0.804**	**0.811**
**Ours**	**0.853**	**0.831**	**0.785**	**0.805**
w/o common augmentation	0.849	0.817	**0.785**	0.800
w/o ICD tree structure	0.835	0.801	0.771	0.786
w/o diagnostic exclusivity	0.821	0.770	0.762	0.764

As shown in Supplementary Table 5, our proposed model outperformed traditional deep learning baselines. BERT-based methods outperformed LSTM-based and BIMPM approaches, as LSTM struggled to learn diagnostic comparison knowledge from limited labeled data, given the vast range of diagnoses requiring comparison.

GPT3.5-Turbo and Llama3-8B were tested using a 4-shot approach, but performed poorly on this task, significantly below fine-tuned models. This may be due to the

task's complexity, making it difficult for the model to fully understand the requirements through instructions alone. After supervised fine-tuning, Llama3-8B-instruct performed best (F1: 81.1%), leveraging its extensive pre-training (on 15T tokens) and rich medical knowledge. This highlights the need for extensive medical knowledge to effectively compare diagnostic relationships, especially for nuanced classifications like included and secondary.

Although Llama3-8B-instruct-SFT's performance is slightly higher than our model (F1 score improvement of 0.6%), its large number of parameters requires approximately 20GB of GPU memory. This limitation restricts the practical application range of this model to deploy in medical institutions. Therefore, considering real-world constraints, we still consider our model as the primary implementation method for now.

As shown in Supplementary Table 5, our contrastive data construction method improves the performance of the pre-training model. Notably, the method based on diagnostic exclusivity in discharge diagnosis list has the greatest impact (2.2%), while common text data augmentation methods offer minimal improvement(0.5%). The ablation results confirm that, using the same fine-tuning data, our method effectively provides the pre-training model with additional expert knowledge, thereby improving its performance.

2.3 Case study of contextual validation

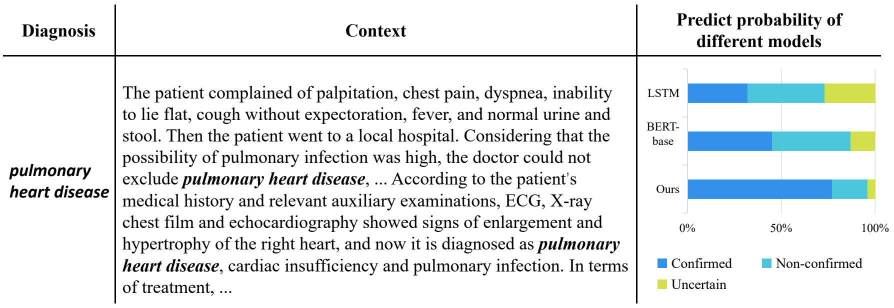

Supplementary Figure 4 Case study of contextual validation. The content translated from Chinese EMR.

Supplementary Figure 4 illustrates a case of pulmonary heart disease in which two contextual descriptions convey different meanings: the one expresses uncertainty during an earlier hospital visit, while the other confirms the diagnosis following examination. This scenario challenges the model to reason over complex clinical logic and correctly associate each diagnosis statement with its corresponding context. The prediction probabilities on the right side of the Supplementary Figure 4 show that LSTM struggles with context comprehension, resulting in uncertainty with evenly distributed classification probabilities. In contrast, the BERT-base model demonstrates better overall context understanding, showing a higher probability of confirmation. Our model, enhanced by prior knowledge-based data augmentation, accurately predicts the

diagnosis as confirmed. In this case, we speculate that identifying location of diagnosis in the context (LOC feature) played a critical role in our model's decision-making.

2.4 Case study of diagnosis deduplication

Supplementary Table 6 Case of diagnosis deduplication.

**Case 1**: Character level	
Diagnosis Pair	Head fracture, skull fracture
Ground Truth	Inclusion (prior), Similar
Edit Distance	Predicted probability of Similarity(50%)
LSTM	Predicted probability of Similarity(98.5%)
BERT-base	Predicted probability of Inclusion(98.5%)
Ours	Predicted probability of Inclusion(99.1%)
**Case 2**: Semantic level	
Diagnosis Pair	posterior circulation cerebral ischemia, cerebral ischemia
Ground Truth	Inclusion (prior), Similar
Edit Distance	Predicted irrelevance
LSTM	Predicted probability of Inclusion(55.3%)
BERT-base	Predicted probability of Inclusion(77.6%)
Ours	Predicted probability of Inclusion(99.3%)
**Case 3**: Knowledge level	
Diagnosis Pair	left hydronephrosis, Abnormal kidney function
Ground Truth	Secondary (prior), Similar
Edit Distance	Predicted Irrelevance
LSTM	Predicted Irrelevance
BERT-base	Predicted Probability of Secondary(44.7%)
Ours	Predicted Probability of Secondary(96.8%)

Note: Diagnosis name translated from Chinese

Supplementary Table 6 presents examples with prediction probabilities for correct predictions. For simple cases (character level), like the relationship between head fracture and skull fracture, all methods predict correctly. For cases (semantic level) requiring character and semantic judgment, semantic-based models perform better, with our model showing greater advantages (higher probability) than BERT and LSTM. For challenging cases (knowledge level) with no character similarity, requiring diagnostic and knowledge-based understanding, even BERT-based methods struggle with limited fine-tuning. However, our method effectively addresses such cases through contrastive training.

3 Supplementary Analysis of Overall Experiments

3.1 DRG version

The CHS-DRG (China Healthcare Security Diagnosis Related Groups), developed in 2019 under the leadership of China's National Healthcare Security Administration, serves as a standardized diagnosis related group (DRG) payment system for nationwide healthcare reimbursement. Its objectives include unifying medical insurance payment standards, optimizing healthcare resource allocation, and controlling unreasonable cost

growth. Based on internationally recognized DRG principles, CHS-DRG incorporates localized adaptations tailored to China's clinical practices and medical insurance data characteristics. Our study adopts CHS-DRG 1.1, which comprises 628 DRG groups, classified using the China Medical Insurance ICD-10 (diagnosis codes) from Medical Insurance version 2.0 and ICD-9-CM-3 (surgical codes).

3.2 This process generated DRG groupings adjusted for missed diagnoses

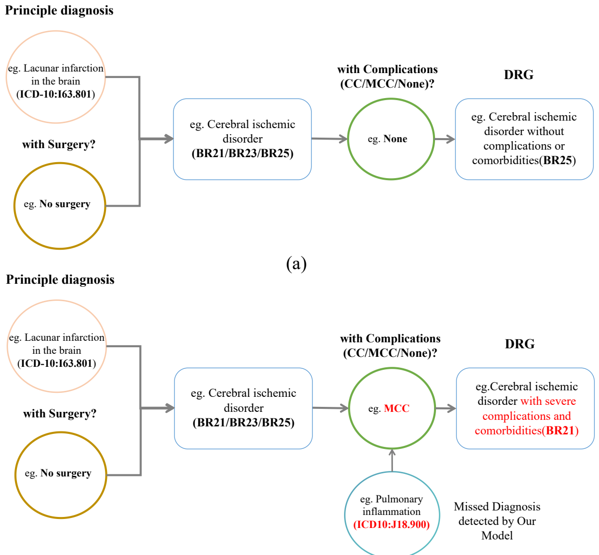

Supplementary Figure 5 Workflow for assessing the impact of missed diagnosis detection on DRG assignment. (a) DRG grouping process. (b) Missed diagnosis detection for correcting DRG grouping.

Input of these data into our missed diagnosis detection system yielded results identifying missed diagnoses, which were then coded using ICD. These diagnoses were evaluated for their status of CC / MCC and compared against the DRG grouping provided by the insurance bureau. Discrepancies in the levels of CC / MCC prompted revisions to the DRG categorizations based on the CHS-DRG scheme. This process generated DRG groupings adjusted for missed diagnoses, as illustrated in the Supplementary Figure 5. The medical record texts in the table were translated from the original Chinese data.

3.3 Coding error analysis in DRG impact assessment

In our DRG-impact analysis (Section 2.4), we identified two types of ICD coding errors: (1) Severe error, where the assigned code does not match the EMRs—these were excluded as incorrect during annotation. For example, the first case in Supplementary

Supplementary Table 7 Error ICD case. Content translated from Chinese EMRs.

Error types	EMR context	Original error ICD	Correct ICD
Severe error	The patient developed mild epigastric pain 3 days prior to admission, which localized to the right lower quadrant after 10 hours, progressing to persistent pain with paroxysmal exacerbations, accompanied by nausea and anorexia. Physical examination and ultrasound findings confirmed acute appendicitis, prompting emergency laparoscopic appendectomy with lysis of intestinal adhesions, which was performed successfully. Postoperative recovery was achieved ...	Intestinal adhesions: K66.002 (CC)	This condition should not be coded.
Minor error	The patient was diagnosed with hilar cholangiocarcinoma based on a one-month history of progressive jaundice and weight loss. Preoperative evaluation revealed pulmonary hypertension (PASP 38 mmHg, Mild), attributed to tumor-related cachexia and chronic hypoxia. Following multidisciplinary team (MDT) discussion, successful biliary stent placement was performed under oxygen support and sildenafil therapy (10 mg three times daily), which effectively stabilized pulmonary pressures. Postoperatively, ...	Pulmonary hypertension: I27.200x012 (MCC)	Mild pulmonary hypertension: I27.200x015 (MCC)

Table 7 involved an incorrect code (intestinal adhesion, K66.002) leading to a false CC designation and subsequent error DRG grouping (this case was excluded from the results). (2) Minor error, where the code is incomplete but does not affect CC/MCC determination or DRG assignment—these were treated as correct during annotation. For the second case in Supplementary Table 7, while coded inaccurately (mild pulmonary hypertension, I27.200x015 coded as pulmonary hypertension, I27.200x012), did not affect CC/MCC determination.

After review, only two instances of the first error type were found (out of 27 correctly grouped DRG cases), suggesting that dictionary-based ICD coding is relatively reliable under the DRG system.

3.4 Comparative analysis of BERT baseline and DKFusion methods

BERT baseline and DKFusion systems adopt identical pipeline architectures and employ the identical diagnosis recall module. They differ fundamentally in their two verify modules implementations: (1) The BERT baseline employs purely native BERT models with standard fine-tuning for both context validation and diagnosis deduplication modules, using the same annotated datasets as DKFusion; (2) In contrast, DKFusion introduces two key innovations: for context validation, it combines prior knowledge encoding with BERT-based contextual encoding through gating fusion (creating an architectural variant of BERT), while for diagnosis deduplication, it enhances a native BERT pretrained model's diagnosis discrimination capability through contrastive pre-training to fusion expert knowledge before fine-tuning (representing a training paradigm variant).

Supplementary Table 8 Comparison of model training methods and data usage

Methods	Contextual validation	Diagnosis deduplication modules
BERT-Based	finetune:<br>9,925 examples (annotated)	finetune:<br>4,999 examples (annotated)
DKFusion	finetune stage1:<br>50,000 examples (data augment)<br>finetune stage2:<br>9,925 examples (annotated)	contrastive pretraining:<br>712,000 examples (data augment)<br>finetune:<br>4,999 examples (annotated)

Comparison of model training methods and data usage. As shown in Supplementary Table 8, we compare the training strategies and data usage between BERT-Based and DKFusion models for both contextual validation filtering and diagnosis deduplication tasks. The BERT-Based approach employs direct fine-tuning using 9,925 labeled samples for contextual validation and 4,999 labeled samples for diagnosis deduplication. In contrast, DKFusion adopts a staged training strategy: for contextual validation, it first performs initial fine-tuning with 50,000 augmented samples followed by secondary fine-tuning with 9,925 labeled samples; for diagnosis deduplication, it conducts continued pretraining with 712,000 augmented samples before final fine-tuning with 4,999 labeled samples.

Comparison of BERT and DKFusion performance. Based on the main experimental results, we focus here on comparing the performance of BERT and DKFusion, with the analysis as follows:

The BERT-based model was obtained by fine-tuning directly on our annotated data. Although it achieves acceptable results on the model-level evaluation, it suffers from severe overfitting due to distributional mismatches and thus degrades substantially on end-to-end experiment of missed diagnosis task. For the contextual validation module, we used a heuristic method based on EMR characteristics for pre-annotation, followed by physician validation, to efficiently and balancedly construct the training dataset. However, this approach may introduce distribution inconsistencies between the module's training data and the end-to-end scenario test data. Specifically, we heuristically treated mentions of discharge diagnoses in clinical contexts as confirmed diagnoses, followed by physician verification. The training set combines data generated from this method with physician annotations from randomly sampled EMR sections, reducing annotation effort while balancing labels. Although this method ensures data quality, there may still be certain contextual style differences between discharge diagnoses and missed diagnoses within EMRs. BERT-based models relying solely on supervised training struggle to effectively overcome this generalization issue. For the diagnosis deduplication module, designed as a four-class classification task to assess relationships between diagnoses, faces challenges with only 4,999 labeled pairs for training. With over 900 million possible pairings under the ICD framework, BERT-based method lacks sufficient diagnosis-relationship knowledge, leading to significant performance drops when handling diverse real-world diagnosis combinations.

The DKFusion model builds on the BERT, going beyond reliance on annotated data by integrating extensive prior and expert knowledge to address the limitations of the vanilla BERT approach. In the contextual validation module, prior knowledge, including LOC, NEG, and ENU features, supplements more task-specific information, while systematic replacement of diagnosis names during training ensures exposure to diverse terminology. For the diagnosis deduplication module, expert knowledge is infused by leveraging the ICD hierarchy and historical EMRs to construct approximately 712,000 diagnosis-pair examples for contrastive pretraining. This large-scale pretraining enriches the model with diagnosis-relationship knowledge, effectively reducing reliance on limited annotated data and alleviating overfitting.

By deeply fusing domain expertise and data-driven augmentation, DKFusion bridges the gap between training distributions and end-to-end evaluation. Thus, DKFusion demonstrates better generalization compared to BERT-based methods across scenario test sets. This advantage confirms that our feature designs and knowledge infusion strategies effectively address the distributional challenges inherent in missed diagnosis detection.

3.5 LLM approach setting

Assume you are a professional physician. Please identify the diagnoses that are not recorded in the <discharge diagnosis> from the following <medical record text>.

1. The recalled diseases must be clearly diagnosed, and it should be determined based on contextual semantics that these diseases are not classified as "suspected," "differential," "knowledge introduction," "excluded," "denied," or any other uncertain or negative conditions.

2. Only recall the confirmed diagnoses that are not listed in the <discharge diagnosis>. If the recalled diagnosis shares any inclusion, similarity, or identical aspect with any diagnosis in the <discharge diagnosis>, it should not be recalled. For instance, if "pneumonia" is already listed, "pulmonary inflammatory changes" should not be recalled.

3. The recalled diagnoses must be the same as those in the original text; do not modify the content.

4. The recalled diagnoses must be explicitly present in the original text, without modifying the content.

5. Conclude your response with: The unrecorded confirmed diagnoses are:

Section-level(text): 1. XX.\n2. XX...

EMR-level(json): [{"diag": "","section": ""}]

<medical record text>:{}

<discharge diagnosis>:{}

[Instruction]
[Requirements]
[Format]
[Input]
[Responses]

[Reasoning process...]
The unrecorded confirmed diagnoses are:
1. XX.\n 2. XX / [{"diag": "","section": ""}].

Supplementary Figure 6 Prompt used in the LLM approach. All LLMs used the same instruction and requirements; however, different output format requirements were applied for different configurations (EMR-level and section-level), and the prompt for the Agent approach were the same as those for the EMR-level configuration.

When setting the instructions for the LLM approach, we also took into account the filtering of both unconfirmed diagnoses and those already recorded in the discharge diagnosis. The specific instructions are shown in Supplementary Figure 6.

3.6 Comparison of the model's precision across different departments

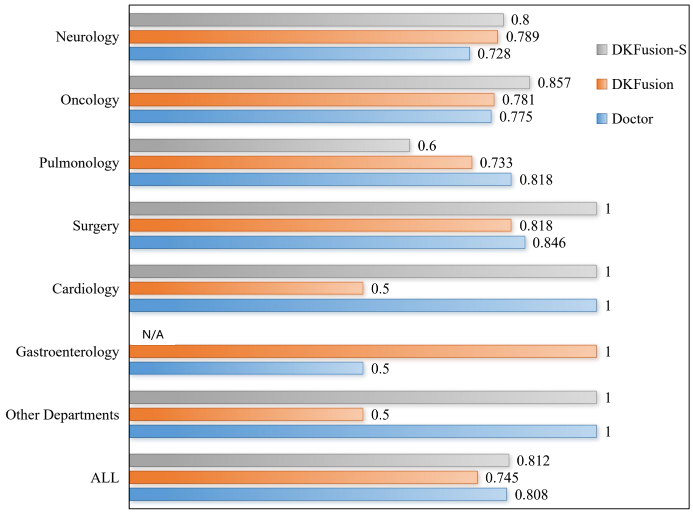

Supplementary Figure 7 Comparison of the model's precision across different departments. This comparison focuses on the precision scores of DKFusion, DKFusion-S, and the physician baseline on the in-domain test set.

Supplementary Figure 7 provides a detailed departmental comparison of precision between DKFusion, DKFusion-S and physicians, revealing that: DKFusion-S outperforms physicians in 3 departments, underperforms in 1 department, and matches physician performance in 2 departments. DKFusion-S achieves marginally higher precision than physicians (by 0.04%) on the overall in-domain test set (HB).

3.7 Correlation analysis of cross-hospital variations

Supplementary Table 9 Comparison of Metrics across Datasets

Dataset	F1 Score	R_{rep}	C_{field}	R_{neg}	C_{diag}
**AH**	**65.4%**	7.5%	9.85	0.45%	55.5%
**JS**	**59.1%**	28.6%	10.61	2.00%	55.0%
**SX-S**	**65.0%**	17.6%	9.94	0.96%	66.0%
**SX-T**	**50.3%**	22.1%	13.08	0.89%	73.7%
**ZJ**	**58.1%**	9.1%	13.48	1.25%	53.5%

To investigate the underlying causes of performance variability across external validation centers, we performed a correlation analysis on EMR datasets from five

out-of-domain test sets (AH, JS, SX-S, SX-T, ZJ). We hypothesized that specific documentation habits and data complexity factors—beyond simple data volume—directly influence model generalization.

We extracted four key metrics to profile the data quality and complexity for each hospital: Field Completeness (C_{field}): The average number of non-empty sections per EMR out of 14 standard sections (e.g., Chief Complaint, History of Present Illness). Repetition Rate (R_{rep}): Proxies the "copy-paste" phenomenon. It is calculated as the average editorial distance ratio between adjacent progress notes. A higher rate indicates high text redundancy and lower information density. Negative Symptom Ratio (R_{neg}): The proportion of negative findings or denied symptoms within the text. High ratios increase linguistic complexity, requiring the model to distinguish between presence and absence of clinical features. Diagnostic Completeness (C_{diag}): The ratio of documented discharge diagnoses to the total ground-truth diagnoses (including missed diagnoses). High completeness implies that clinicians have already documented the obvious cases, leaving only subtle or complex cases as "missed diagnoses" for the model to identify.

By comparing hospitals with distinct performance gaps, we identified specific factors hindering model generalization:

Case 1: Impact of Documentation Redundancy (JS vs. AH) While hospital AH achieved the highest F1 score (65.4%), hospital JS performed significantly lower (59.1%). A key differentiator is the Repetition Rate. JS exhibited a high repetition rate of 28.6% compared to AH's 7.5%. Qualitative inspection suggests that JS records frequently contain copy-pasted progress notes with minimal updates, diluting information density. Additionally, JS had a higher Negative Symptom Ratio (2% vs. 0.45%), introducing linguistic noise that challenges the model's false-positive identification capabilities.

Case 2: Impact of Field Relevance (SX-T vs. SX-S) Surprisingly, higher Field Completeness did not guarantee better performance. SX-T had high field completeness (13.08) but the lowest F1 score (50.3%), whereas SX-S had lower completeness (9.94) but higher performance (65.0%). Our analysis shows that datasets like SX-S and AH consistently lack subjective fields such as "Characteristics of patient," "Diagnostic Basis," and "Differential Diagnosis." Paradoxically, the absence of these fields may have reduced noise, as these sections—particularly "Differential Diagnosis"—often contain complex clinical reasoning and false-positive cases that challenge the model's understanding. Conversely, the "complete" documentation in SX-T introduced more irrelevant variance, complicating the extraction task. Furthermore, Diagnostic Completeness emerged as a critical influencing factor(73.7% in SX-T vs. 66.0% in SX-S), reflecting clinicians' emphasis on avoiding missed diagnoses. This resulted in subtler and complex instances of missed diagnoses in such datasets, making them harder for the model to identify. While both EMR section completeness and diagnostic completeness reflect data quality, they also represent the complexity of EMR, demanding greater model generalization capability.

Since the relationship between individual factors and model performance is complex and difficult to measure accurately, we normalized these four factors to a scale of [0, 1] and calculated a Composite Complexity Score (S_{comp}) for each hospital dataset:

$S_{comp} = \text{Norm}(R_{rep}) + \text{Norm}(C_{field}) + \text{Norm}(R_{neg}) + \text{Norm}(C_{diag})$ We then analyzed the correlation between S_{comp} and the model's F1 score. As shown in Supplementary Figure 9, there is a negative correlation (Pearson $r = -0.8424$) between the Composite Complexity Score and model performance.

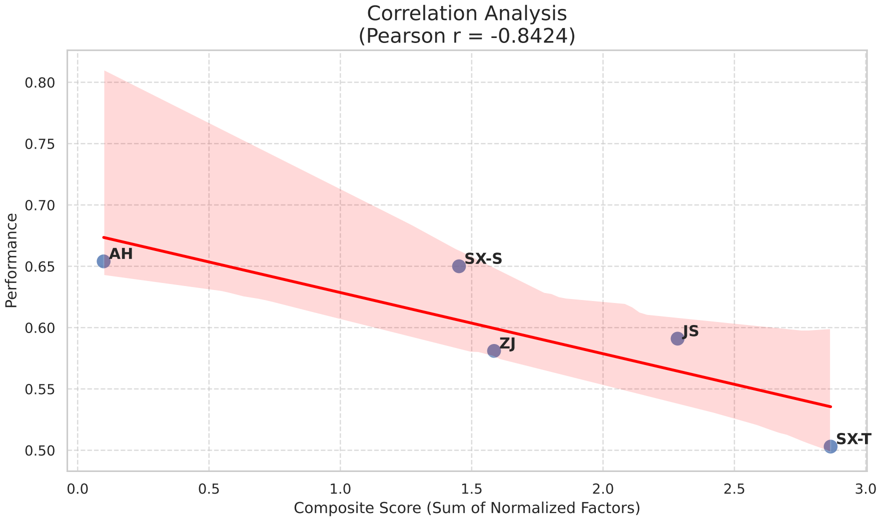

Supplementary Figure 8 Correlation analysis between EMR Composite Complexity Score and Model Performance (F1). The red line indicates the linear fit, showing that higher data complexity/noise is strongly correlated with lower performance.

Conclusion: The variation in external validation performance is not random but is driven by EMR quality, writing styles, and the completeness of discharge diagnoses. Specifically, high rates of text repetition (e.g., copy-paste practices), linguistic complexity (such as negative symptom reporting), and the presence of complex sections combined with stricter missed-diagnosis control protocols in EMRs represent concrete factors that degrade model performance across institutions.

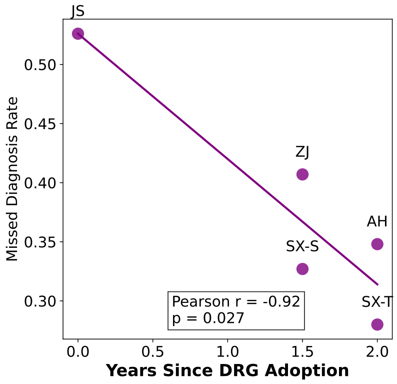

Supplementary Figure 9 Correlation between years since DRG adoption and predicted missed diagnosis rates. The duration each hospital has implemented the DRG payment system (measured in 0.5-year units) prior to data collection. Pearson correlation coefficients (r) and p-values are annotated.

We also analyzed the relationships among years of DRG adoption and missed-diagnosis rates. DRG adoption duration was determined from public reports, using 0.5-year increments as the minimum unit. The results suggested a strong negative correlation (Pearson r = -0.92, p = 0.027) between years of DRG adoption and missed diagnosis rates (Supplementary Figure 9). Despite the small sample size, the statistical significance and high correlation coefficient suggest that longer exposure to DRG-based payment systems is closely associated with lower missed diagnosis rates.

3.8 Sensitivity analysis of module thresholds

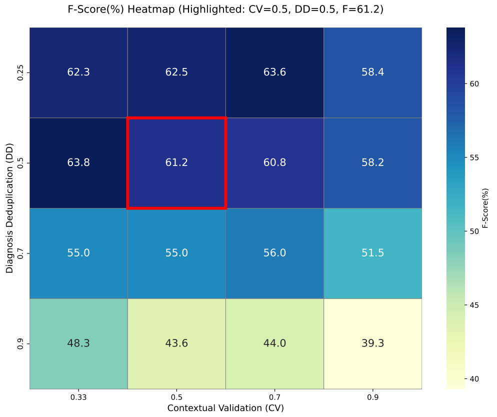

Supplementary Figure 10 Sensitivity analysis of decision thresholds (Grid Search). The heatmap illustrates F-score variations on the test set across different probability thresholds for the Contextual Validation and Diagnosis Deduplication modules. The red box highlights the selected standard threshold of 0.5 for both modules, yielding an F-score of 61.2%.

To examine threshold selection and sensitivity of Contextual Validation and Diagnosis Deduplication modules, we conducted a grid search experiment on the test set. We evaluated the variation in F-score performance by adjusting the decision thresholds for the Contextual Validation (CV) module ranging from [0.33, 0.9] and the Diagnosis Deduplication (DD) module ranging from [0.25, 0.9]. The results are visualized in the Heatmap of Supplementary Figure 10.

Based on the analysis, we found that: (1)Sensitivity to CV Thresholds (X-axis): The model demonstrates stable performance at lower to moderate confidence thresholds (0.33–0.7). For instance, with DD fixed at 0.5, increase the CV threshold from 0.33 to 0.7 results in a less F-score change (63.8% to 60.8%). However, strictly increasing the threshold to 0.9 leads to a significant performance drop. This indicates that an overly strict validation threshold excludes potential correct diagnoses, thereby reducing recall. (2)Sensitivity to DD Thresholds (Y-axis): Similarly, the deduplication module performs best with thresholds between 0.25 and 0.5. Performance degrades notably when the threshold is raised to 0.7 or 0.9 (e.g., dropping to 55.0% and 43.6% at CV=0.5). This suggests that the model effectively identifies redundancies (Similar/Including categories) at standard probability thresholds, while higher thresholds exert excessive constraints, hurting overall performance. (3)Justification for Selected Thresholds (0.5/0.5): As shown in the red box in the figure, our pre-selected thresholds of 0.5 for both CV and DD yield an F-score of 61.2%. While a slightly higher score (63.8%) was observed at (CV=0.33, DD=0.5), the performance at (0.5/0.5) lies within the robust high-performance region (top-left quadrant). We chose 0.5 as it represents the standard, intuitive decision boundary for probability-based classification tasks (Softmax), balancing Precision and Recall without "data snooping" or overfitting to the specific test set distribution.

Supplementary References

[1] Wang, Z., Hamza, W., Florian, R.: Bilateral multi-perspective matching for natural language sentences. arXiv preprint arXiv:1702.03814 (2017)
